# Salt stress in olive tree shapes resident endophytic microbiota

**DOI:** 10.3389/fpls.2022.992395

**Published:** 2022-09-29

**Authors:** Federico Vita, Leonardo Sabbatini, Fabiano Sillo, Stefano Ghignone, Marzia Vergine, Werther Guidi Nissim, Stefania Fortunato, Anna Maria Salzano, Andrea Scaloni, Andrea Luvisi, Raffaella Balestrini, Luigi De Bellis, Stefano Mancuso

**Affiliations:** ^1^ Department of Biology, University of Bari Aldo Moro, Bari, Italy; ^2^ Department of Agriculture, Food, Environment and Forestry (DAGRI), University of Florence, Florence, Italy; ^3^ National Research Council of Italy, Institute for Sustainable Plant Protection (CNR-IPSP), Torino, Italy; ^4^ Department of Biological and Environmental Sciences and Technologies, University of Salento, Lecce, Italy; ^5^ Department of Biotechnology and Biosciences, University of Milano-Bicocca, Milano, Italy; ^6^ Proteomics, Metabolomics and Mass Spectrometry Laboratory, National Research Council of Italy, Institute for the Animal Production System in the Mediterranean Environment (CNR-ISPAAM), Portici, Italy; ^7^ Fondazione per il futuro delle città (FFC), Florence, Italy

**Keywords:** salt stress, olive tree, microbiota, endophytic community, ngs, metabarcoding

## Abstract

*Olea europaea* L. is a glycophyte representing one of the most important plants in the Mediterranean area, both from an economic and agricultural point of view. Its adaptability to different environmental conditions enables its cultivation in numerous agricultural scenarios, even on marginal areas, characterized by soils unsuitable for other crops. Salt stress represents one current major threats to crop production, including olive tree. In order to overcome this constraint, several cultivars have been evaluated over the years using biochemical and physiological methods to select the most suitable ones for cultivation in harsh environments. Thus the development of novel methodologies have provided useful tools for evaluating the adaptive capacity of cultivars, among which the evaluation of the plant-microbiota ratio, which is important for the maintenance of plant homeostasis. In the present study, four olive tree cultivars (two traditional and two for intensive cultivation) were subjected to saline stress using two concentrations of salt, 100 mM and 200 mM. The effects of stress on diverse cultivars were assessed by using biochemical analyses (*i.e.*, proline, carotenoid and chlorophyll content), showing a cultivar-dependent response. Additionally, the olive tree response to stress was correlated with the leaf endophytic bacterial community. Results of the metabarcoding analyses showed a significant shift in the resident microbiome for plants subjected to moderate salt stress, which did not occur under extreme salt-stress conditions. In the whole, these results showed that the integration of stress markers and endophytic community represents a suitable approach to evaluate the adaptation of cultivars to environmental stresses.

## Introduction

Olive tree (*Olea europaea* L.) is a long-living, evergreen species that has been an historically important sclerophyll plant in the Mediterranean basin, considering that olives represent a food and oil source for centuries in all the countries around the Mediterranea sea (Diez et al., 2015; Besnard et al., 2018). Olive tree is considered a rustic plant adapted to a marginal life, especially in coastal areas characterized by prolonged summer drought and elevated soil salinity (Gucci et al., 1997). Currently, salinization of soils represents an important threat to crop production in the Mediterranean basin so far (Maggio et al., 2011), also affecting olive tree production. Essentially, olive trees grown on salinized show growth reduction, shortened internodes, small leaves with thickened mesophyll and cell walls, reduced blooming, decreased pollen germinability and number of fruits (Gucci et al., 1997). The main visible symptoms of salinity are represented by leaf chlorosis and necrosis, desiccation of flowers and new shoots, and leaf abscission after a long stress exposure. Therefore, premature leaf drop can be the last defense mechanism against high salt concentrations, thereby allowing the reduction of toxic ion uptake and transpiration rate at the whole plant level (Loupassaki et al., 2002). Unfortunately, the onset of symptoms is not an efficient diagnostic method to determine the high salt concentration in the soil, because the damaged entity is very similar to other stress symptoms (*i.e.*, nutritional deficiencies and drought stress). Several studies have shown that the olive tree’s ability to respond to high salt concentrations is closely related to an effective mechanism of ions exclusion and retention by the roots system (Chartzoulakis, 2005; Tattini et al., 2008).

However, the ability of different olive tree cultivars to withstand salinity does not rely only on different K^+^/Na^+^ ratios in different tissues but is due to the interaction among several physiological, metabolic, and molecular factors. Recently, several researchers have endeavored to encompass the relationship between plants and resident microorganisms, mainly endophytic bacteria, which can help plants to alleviate the severity of many abiotic stresses. Although endophytic bacteria communities residing inside plant tissues sometimes show neutral effects, in many cases they may be beneficial for the plant by promoting growth and mineral uptake from the soil, nitrogen fixation, and siderophore production, thus mitigating biotic or abiotic stresses (Lodewyckx et al., 2002). Although ethylene, produced by plants grown in salt stressed soils, can play a positive regulatory role in salt stress tolerance (Riyazuddin et al., 2020), elevated ethylene levels have been reported to have adverse effects on salinity tolerance (Chen et al., 2014). Several studies have verified that some endophytes belonging to the genera *Arthrobacter*, *Bacillus*, *Isoptericola* and *Streptomyces* showed the ability to produce 1-aminocyclopropane1-carboxylate (ACC) deaminase, which catalyzes the conversion of the ethylene precursor ACC to ammonia and α-ketobutyrate (Qin et al., 2016). Furthermore, endophytes belonging to genera *Bacillus*, *Halomonas*, *Kushneria*, and *Micrococcus* can improve tolerance of plants to salt stress inducing changes in plant antioxidant enzyme activities, for example, ascorbate peroxidase (APx), catalase (CAT), guaiacol peroxidase (GPx), and superoxide dismutase (SOD) (Navarro-Torre et al., 2017).

The deciphering of resident microbial communities and their role in tree physiology is a rising topic (Hacquard and Schadt, 2015; Blair et al., 2018; Cregger et al., 2018; Leopold and Busby, 2020). A comprehensive knowledge of microbial communities associated with the root system, including the root endosphere and the rhizospheric soil, has been recently reported by Fernández-González et al. (2019). Regarding the endophytic/epiphytic composition in the olive trees, recent studies have shown that differences emerge when different cultivars (Mina et al., 2020) and diverse European origin areas (Müller et al., 2015) are taken into account, or the microbiome profile of plant tissues is comparatively evaluated (Malacrinò et al., 2022). The microbiota of olive roots across different seasonal patterns was also elucidated (Chialva et al., 2021). Thus, understanding the complex mechanisms of woody plants, including important crop species such as olive tree, and their relationship with endophytic microorganisms could open new avenues for alternative ecological strategies to grow plants in soils affected by high salt concentrations.

Thus, the main aim of this study was to better understand how resident olive leaves endophytic bacterial communities are shaped by different levels of salt stress, considering different olive genotypes. In this view, biochemical and metabarcoding data were integrated with the goal of trying to explain how four olive tree cultivars subjected to different saline concentrations cope with stress. Results can represent a step forward in deciphering the role of microbiota in facing plant abiotic stresses in a woody crop plant.

## Materials and methods

### Experiment design and samples collection

A set of four olive tree cultivars own-rooted were chosen: Frantoio (FR), Lecciana (LA), Leccino (LE) and Oliana (OL) were selected based on data already available in literature (see below).

LA and OL cultivars were purchased from Agromillora nursery (Barcelona, Spain), meanwhile, FR and LE were obtained from Vivai Pietro Pacini nursery (Pescia, Italy). Plants (self-rooted cuttings) of 1-year old were placed in 8x8 cm pots.

LA and OL are Italian and Spanish olive cultivars, respectively, used worldwide for super high-density olive cropping systems (SHD) and are characterized by medium and low vigorous, respectively. Particularly, LA has been developed as a new cultivar since 1998, generated by crossing between Arbosana and Leccino cultivars (Camposeo et al., 2021). FR cultivar is an Italian cultivar but is widely cultivated in all parts of the world, including Chile, Argentina, South Africa, Pakistan and China. It is a vigorous cultivar appreciated for the constant and good productivity of the fruits. Last, LE is an Italian cultivar characterized by medium vigor and constant yield and showing a similar distribution to FR.

Diverse salt stress tolerance of FR and LE cultivars have been reported by Tattini et al. (2008) and Cimato et al. (2010), suggesting that FR is much more tolerant than LE. To validate these already published data, LE and FR cultivars were selected, in addition to LA and OL, to define a salt tolerance range within the group of selected cultivars. Forty-five rooted cuttings for each cultivar were transplanted in new black pots (8 x 8 x 18 cm) containing approximately 1,152 dm^3^ of sterilized perlite substrate (Agrilit 3, Agriperlite Italiana, Alzaia Trento, Italy). At transplanting all plants showed only the main stem that was approximately 40-60 cm long. Plants were grown in a greenhouse from June to September 2019 with an ambient light (500 µmol m^-2^ s^-1^ PAR), mean temperature 28°C (max 34.5°C – min 24.9°C), mean humidity 46% (max 60.5% - min 34.4%) and a photoperiod of 15 h light/9 h dark, at the University of Florence (Italy) (lat. 43°48′58.6″ N, long. 11°11′58.1″ E). The experimental layout is reported in **Supplementary Figure 1**. Five plants for each cultivar were placed on three benches each one equipped with a closed recirculating irrigation system. The “ebb-and-flood” bench system used in this trial allowed a complete automatized regulation of the solution depth and the exposure time, which, in the current trial, were set 4 cm and 15 min respectively. One month after transplanting (to give plants the time to acclimatize in the new growth environment), based on the currently available information for this species, plants were subjected to three different salt concentration treatments, with 15 plants per treatment (45 plants for each cultivar in total): control (T1), plants irrigated with Hoagland ½ solution (Hoagland et al., 1950); treatment 1 (T100), plants irrigated with 100 mM of NaCl in Hoagland ½ solution; treatment 2 (T200), plants irrigated with 200 mM of NaCl in Hoagland ½ solution (Hoagland et al., 1950). All solutions were prepared using distilled water. A sampling have been also done before applying salt stress (T0). According to Abdallah et al. (2018), to reduce the risk of osmotic shock, the final NaCl concentrations were gradually reached by adding 50 mM NaCl every two days until the final 200 mM concentration was reached. Every week, soil electrical conductivity (1542.08 µS cm-1/6.8 for control, 10.44 mS cm-1/6.8 for 100mM, and 19.07 mS cm-1/6.8 for 200mM) and pH were checked with portable conductimetry and corrected, whenever necessary, with HNO_3_. Additionally, when necessary, water was added until it reached the volume of 300 L, to maintain the optimal volume of each of the three used tanks (one per treatment). The treatments were carried out for about 70 days until the first stress salinity symptom (*i.e.*, cellular necrosis) was evident on the apical leaves in T200 plants (**Supplementary Figure 2**). To verify the status of the used plants before each sampling step (T0 and T1), biometric measurements have been carried out (**Supplementary Figures 3, 4**, **S4**). Leaves already formed at the beginning of the experiment were collected for the subsequent biochemical and metabarcoding analyses. Plants were randomly selected for the diverse analyses and leaves were selected among those above the three basal internodes (leaves of one-year old) and avoiding the apical part (the part developed during the experiment), collecting the leaves at the level central internodes (leaves already developed less than one-year old).

### Chlorophyll and proline analysis

Spectrophotometric analyses were performed using 10 mg of olive fresh leaves, which were collected from six different plants (n=6) for each cultivar and treatment, at the end of the experiment. Leaf samples were collected from the middle of the leaf main axis and then frozen in liquid nitrogen to obtain a fine powder. Afterwards, 1 mL of cold methanol was added, and samples were shaken for 30 min and then centrifuged at 10000 x *g* for 10 min. The supernatant was collected and used to read the absorbance at 665, 652, and 470 nm for Chlorophyll a (Chla), b (Chlb) and carotenoids, respectively. The absorbance was read using a TECAN spectrophotometer through a 96 black multi-plate reader. Pigment quantification was done using the equations reported in Wellburn (1994).

For proline determination, 200 mg of fresh leaves were collected (n=5) and frozen with liquid nitrogen before to add 1.5 mL of 3% w/v sulfosalicylic acid. Samples were vortexed and then centrifuged at 14000 x *g* for 15 min. Then, the samples were centrifuged using a benchtop centrifuge with maximum speed, and the supernatant was collected in glass tubes. The reaction mixture was prepared by warming 1.25 g ninhydrin in 30 mL glacial acetic acid and 20 mL of 6 M phosphoric acid, then vortexed until dissolved. Then, 0.3 mL of buffer was added to the extracted supernatant, and the glass tubes were then heated at 100°C for 1 h. Following this step, color of the samples turned red-violet, depending on proline concentration. Next, the samples were cooled on ice to stop the reaction. Subsequently, samples were extracted by 1 mL of toluene and vortexed to allow the separation of organic and water phases. Samples were then analyzed with a spectrophotometer (Biorad SmartSpec Plus, Hercules, California, USA), and the absorbance was read at 520 nm wavelength using toluene as blank. The calibration curve was made with a standard compound at different concentrations, and proline concentration in relation to fresh weight was calculated as already reported (Bates et al., 1973).

### DNA extraction and sequencing – NGS

Leaf samples were collected at the beginning (T0) and the end of the experiment (Control T1 and salt-treated T100, T200). DNA from olive leaves (3 biological independent replicates, *i.e.*, 3 from 3 plants, n=3) was extracted as described by Edwards et al. (1991), with minor modifications. First, about 1 gram of leaves was transferred into extraction bags (BIOREBA, Switzerland), and 4 ml of 0.2 M Tris–HCl pH 9, 0.4 M LiCl, and 25 mM EDTA (Tris-HCl-based extraction buffer) was added. According to Vergine et al. (2019), samples were then homogenized using a semi-automatic homogenizer. The DNA solution was first extracted with phenol-chloroform-isoamyl alcohol (25:24:1 ratio) to remove protein contaminants; then, DNA was precipitated with isopropanol. The isolated DNA was then quantified and used as a template for PCR amplification with primers CS1-341F and CS2-806R (Caporaso et al., 2011; Klindworth et al., 2013) the V3-V4 variable regions of the 16s rDNA gene. A mixture of PNA (peptide nucleotide acid) blocker oligos (PNA Bio Inc., USA) was added to increase the sequencing process’s accuracy by avoiding amplification of chloroplast and mitochondria sequences. Reads were collected as a couple for each sample (Paired-End reads) for each condition. A total of 48 libraries were generated from the starting samples, 12 for T0 (Control) and 36 for T1 (Control, T100 and T200). Amplicons were sequenced using an Illumina MiSeq platform (v3 chemistry) at the Génome Québec Innovation Center, McGill University (Montréal, Canada).

### Assessment of microbial communities by QIIME2 pipeline

Paired-end sequences spanning the V3–V4 regions of the bacterial 16S rRNA were initially analyzed using QIIME2 v. 2021.4 (Quantitative Insights Into Microbial Ecology 2). Reads were imported into the QIIME2 environment and quality checking/filtering was performed. Adapter sequences were removed using cutadapt (Martin, 2011), and sequences were truncated and 280 (forward library) and 200 (reverse library) bases from the start. Sequences were denoised using dada2 (Callahan et al., 2016) already included in QIIME2. Clustering was performed with VSEARCH cluster-features-*de-novo* (–p-perc-identity 0.97) (Rognes et al., 2016). Sequences were then classified using a pre-trained naïve Bayes classifier (silva-138-99-nb-classifier.qza) according to sklearn feature classifier method (Pedregosa et al., 2011). Output from QIIME2 (ASV table, taxonomy, metadata file and tree) were then imported in R using the qiime2R package v0.99.34 (Bisanz, 2018) to generate a phyloseq object (McMurdie and Holmes, 2013). In particular, the diversity within samples (α-diversity) was calculated using Chao1, Shannon and Simpson indexes, whereas the diversity among samples (β-diversity) was calculated by both Bray Curtis (Beals, 1984) and weighted Unifrac (Chang et al., 2011), and then visualized in a two-dimensional principal coordinates analysis (PCoA) according to different variables using the ampvis2 package (Albertsen et al., 2015). According to Bray Curtis and Jaccard distances, network analyses were also performed using the package phyloseq (McMurdie and Holmes, 2013).

A differential heat tree was depicted using the package metacoder (v. 0.3.5) (Foster et al., 2017) to graphically display the most abundant taxa among treatments. Outputs from QIIME2 (ASV table, taxonomy and metadata files) were then used with MicrobiomeAnalyst (Chong et al., 2020) for data assessment. First, reads were processed by setting “5” as minimum count of features (10% of prevalence in samples) and 10% of features with low variance in samples based on IQR. Then, libraries were rarefied as reported using the minimum sample size. Finally, results were reported according to univariate analysis (FDR cutoff = 0.05), linear discriminant analysis Effect Size (LEfSe) (Segata et al., 2011) and random forest analysis (Liaw et al., 2002).

### Statistical analysis

Data concerning chlorophyll and proline concentrations were first submitted to a Shapiro-Wilk test for checking their normality. Datasets showing p values below the threshold of 0.05 were log-transformed prior to proceeding with further statistical analyses. Two-way analysis of variance was used to determine significant cultivar and treatment effects as well as interactions for all data. All data were statistically analyzed using two-way ANOVA test using SPSS version 24 software (IBM^®^ Armonk, USA). The significant differences were evaluated *post-hoc* using Tukey’s HSD test with a level of significance of *p* < 0.05. Statistical assessment of metabarcoding data is included in the specific paragraphs (see above).

## Results

### Chlorophyll, carotenoid and proline contents

**Figure 1:**
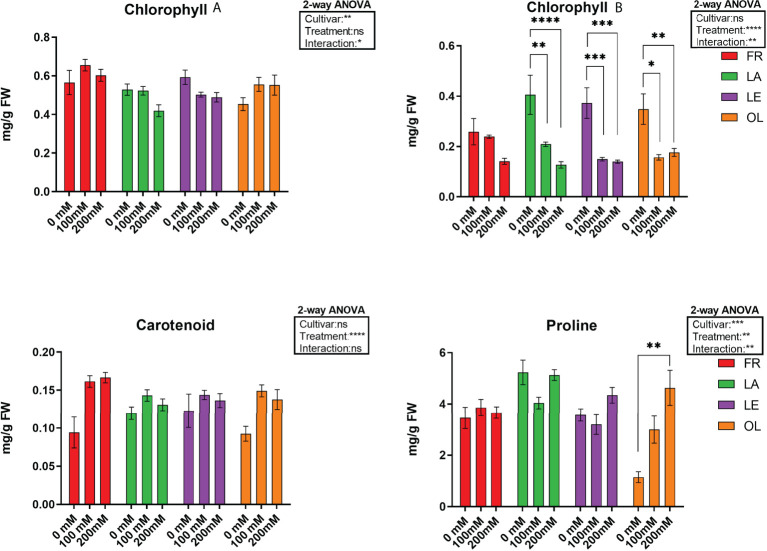
Pigment, chlorophyll A (Chla), clorophyll B (Chlb) and carotenoids) and proline data, and corresponding results from 2-way ANOVA analysis. Data are the mean values consisting of six (n = 6) and five (n = 5) independent replicates for pigments and proline analyses, respectively, for each experimental condition. *Post-hoc* tests were performed according to cultivar (Chla, Chlb and proline) and treatment (Carotenoid) variable. **** < 0.0001 *** < 0.001 ** < 0.01 * < 0.05.

Pigment analyses (**Figure 1**, **Table 1**) showed that cultivars reacted differently in response to stress. Differences were detected mainly in Chlb and carotenoid contents, whereas Chla content was not significantly affected by the stress, despite a slight decrease reported in two (LA, LE) out of four cultivars. In this view, Chlb content decreased due to salt treatment, mostly in the LA, LE, OL T200, whereas the decrease reported in FR T200 was not statistically significant. Furthermore, the different stress treatment did not affect the Chlb content since no significant differences were detected between T100 and T200 in LE and OL, with a slight decrease occurring in FR and LA (T200). About the ratio between Chla/Chlb, T200 was significantly different from T0 in all the considered cultivars while T100 was significantly different from T0 only for LE and OL (**Supplementary Figure 5**, **Table 1**). Conversely, the carotenoid content slightly increased in treated samples compared to control, with a significant increase in FR and OL. Proline content was quantified to highlight the response of a compatible osmolyte to salt stress. Results indicated that only the OL cultivar displayed a clear response pattern, with a significant increase of proline content in T200 compared to control. By contrast, the other cultivars did not show any consistent change in response to stress.

**Table 1:** *Post-hoc* test results of two-way ANOVA analyses on pigment data, considering the Cultivar factor, with exception of Carotenoid data where Treatment factor was considered.

Tukey’s multiple comparisons test	Predicted (LS) mean diff.	95,00% CI*	Significant?	Summary	Adjusted P-Value
***Chlorophyll a***					
**FR**					
0 mM vs. 100mM	-0.09017	-0,2618 to 0,08148	No	ns	0.8181
0 mM vs. 200mM	-0.03755	-0,2176 to 0,1425	No	ns	0.9999
100mM vs. 200mM	0.05262	-0,1274 to 0,2326	No	ns	0.9973
**LA**					
0 mM vs. 100mM	0.005774	-0,1659 to 0,1774	No	ns	>0,9999
0 mM vs. 200mM	0.1093	-0,06237 to 0,2809	No	ns	0.5795
100mM vs. 200mM	0.1035	-0,06814 to 0,2751	No	ns	0.6573
**LE**					
0 mM vs. 100mM	0.09	-0,08164 to 0,2616	No	ns	0.8198
0 mM vs. 200mM	0.1037	-0,06796 to 0,2753	No	ns	0.6549
100mM vs. 200mM	0.01368	-0,1580 to 0,1853	No	ns	>0,9999
**OL**					
0 mM vs. 100mM	-0.1023	-0,2739 to 0,06936	No	ns	0.6734
0 mM vs. 200mM	-0.09876	-0,2704 to 0,07289	No	ns	0.7188
100mM vs. 200mM	0.003531	-0,1681 to 0,1752	No	ns	>0,9999
***Chlorophyll b***					
**FR**					
0 mM vs. 100mM	0.01959	-0,1215 to 0,1607	No	ns	>0,9999
0 mM vs. 200mM	0.1176	-0,02970 to 0,2650	No	ns	0.2406
100mM vs. 200mM	0.09804	-0,04302 to 0,2391	No	ns	0.4387
**LA**					
0 mM vs. 100mM	0.1966	0,04627 to 0,3470	Yes	**	0.0023
0 mM vs. 200mM	0.2787	0,1283 to 0,4290	Yes	****	<0,0001
100mM vs. 200mM	0.08201	-0,05249 to 0,2165	No	ns	0.6345
**LE**					
0 mM vs. 100mM	0.223	0,07267 to 0,3734	Yes	***	0.0003
0 mM vs. 200mM	0.233	0,08259 to 0,3833	Yes	***	0.0001
100mM vs. 200mM	0.009915	-0,1246 to 0,1444	No	ns	>0,9999
**OL**					
0 mM vs. 100mM	0.1913	0,04096 to 0,3417	Yes	**	0.0033
0 mM vs. 200mM	0.1718	0,02144 to 0,3222	Yes	*	0.0129
100mM vs. 200mM	-0.01952	-0,1540 to 0,1150	No	ns	>0,9999
***Carotenoid***					
**0 mM**					
FR vs. LA	-0.02540	-0.08672 to 0.03591	No	ns	0.9547
FR vs. LE	-0.02861	-0.08678 to 0.02956	No	ns	0.8678
FR vs. OL	0.001752	-0.05956 to 0.06307	No	ns	>0.9999
LA vs. LE	-0.003212	-0.06138 to 0.05496	No	ns	>0.9999
LA vs. OL	0.02715	-0.03416 to 0.08847	No	ns	0.9296
LE vs. OL	0.03037	-0.02780 to 0.08854	No	ns	0.8181
**100 mM**					
FR vs. LA	0.01848	-0.03158 to 0.06855	No	ns	0.9803
FR vs. LE	0.01769	-0.03238 to 0.06775	No	ns	0.9859
FR vs. OL	0.01223	-0.03784 to 0.06229	No	ns	0.9994
LA vs. LE	-0.0007932	-0.05086 to 0.04927	No	ns	>0.9999
LA vs. OL	-0.006256	-0.05632 to 0.04381	No	ns	>0.9999
LE vs. OL	-0.005463	-0.05553 to 0.04460	No	ns	>0.9999
**200mM**					
FR vs. LA	0.03579	-0.01671 to 0.08830	No	ns	0.4683
FR vs. LE	0.03024	-0.02226 to 0.08275	No	ns	0.7103
FR vs. OL	0.02874	-0.02376 to 0.08125	No	ns	0.7699
LA vs. LE	-0.005550	-0.05561 to 0.04451	No	ns	>0.9999
LA vs. OL	-0.007050	-0.05711 to 0.04301	No	ns	>0.9999
LE vs. OL	-0.001499	-0.05156 to 0.04856	No	ns	>0.9999
***Proline***					
**FR**					
0 mM vs. 100mM	-0.4053	-2,137 to 1,327	No	ns	0.9995
0 mM vs. 200mM	-0.208	-2,055 to 1,639	No	ns	>0,9999
100mM vs. 200mM	0.1972	-1,722 to 2,116	No	ns	>0,9999
**LA**					
0 mM vs. 100mM	1.194	-0,8950 to 3,283	No	ns	0.6958
0 mM vs. 200mM	0.1046	-2,080 to 2,289	No	ns	>0,9999
100mM vs. 200mM	-1.09	-3,008 to 0,8295	No	ns	0.7042
**LE**					
0 mM vs. 100mM	0.3743	-1,961 to 2,710	No	ns	>0,9999
0 mM vs. 200mM	-0.775	-3,252 to 1,702	No	ns	0.9933
100mM vs. 200mM	-1.149	-2,996 to 0,6972	No	ns	0.5805
**OL**					
0 mM vs. 100mM	-1.849	-4,243 to 0,5440	No	ns	0.2695
0 mM vs. 200mM	-3.487	-6,098 to -0,8751	Yes	**	0.0021
100mM vs. 200mM	-1.637	-3,726 to 0,4519	No	ns	0.2517

*= 95% confidence interval for the difference between two means. **** < 0.0001; *** < 0.001; ** < 0.01; * < 0.05; ns, not significant.

### The endophytic bacteria community associated with olive leaves

**Figure 2:**
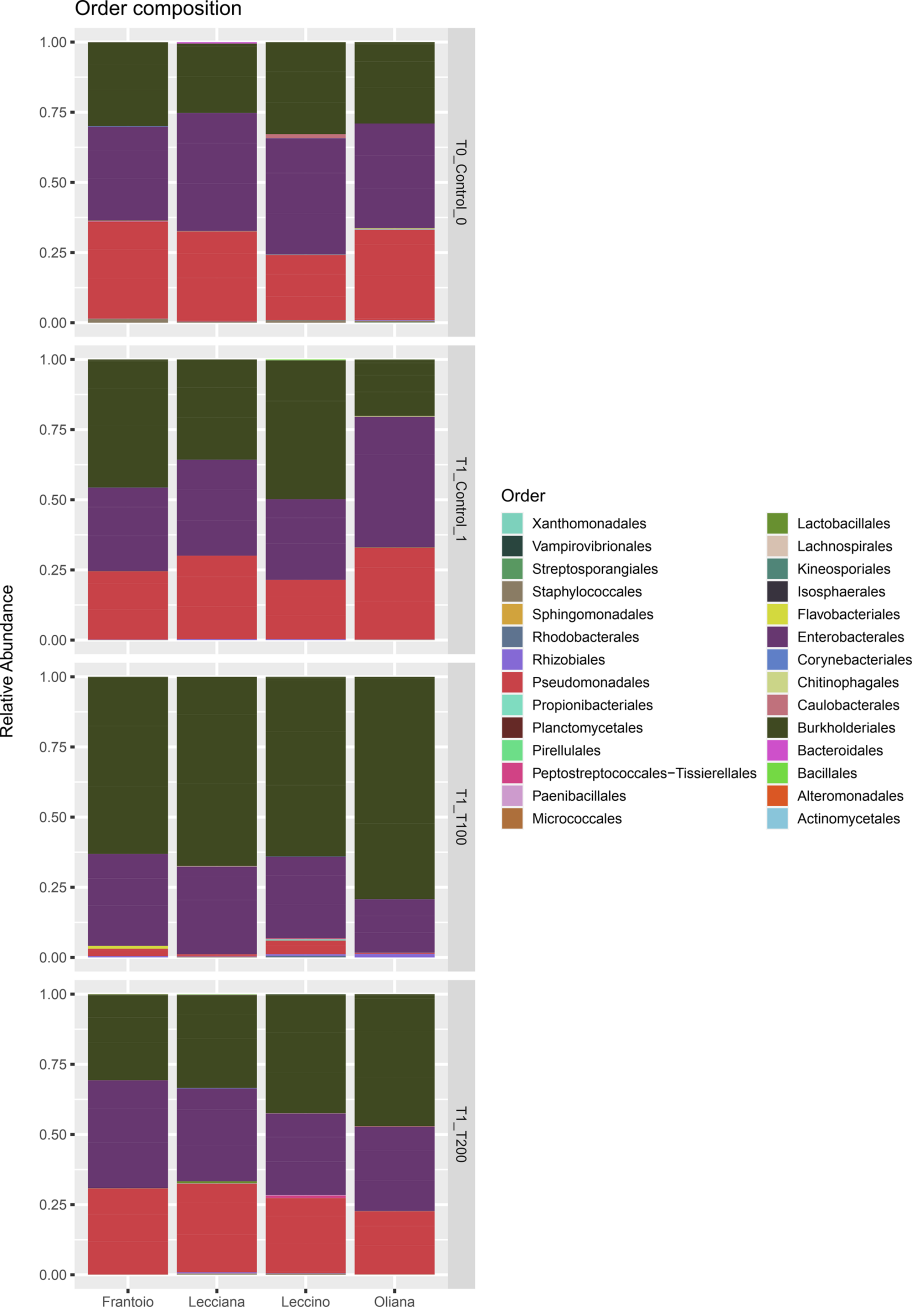
Barplot of the identified ASV at the order level. Relative abundance of detected orders in samples grouped for cultivar and treatment. Represented ASVs were filtered (threshold = 0.5%) to display only significant taxa.

**Figure 3:**
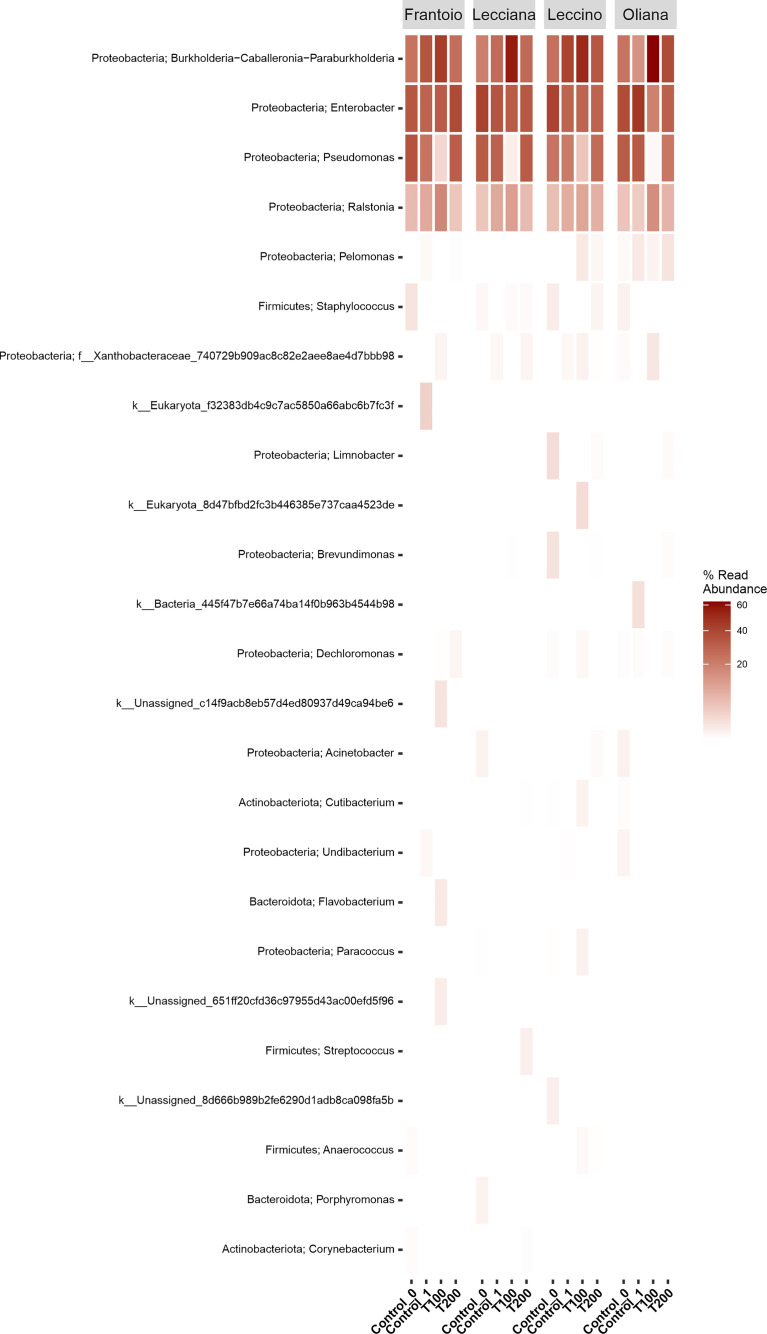
Heat-map of differentially abundant taxa. Data were computed at the genus level using the r package ampvis2. Data were grouped by cultivar and then were classified based on the percentage of read abundance.

A total of 665890 quality- and chimera- filtered bacterial sequences were obtained from the four analyzed cultivars. The average sequence counts per sample was 13872 (total range: from 4738 to 32725). Library size and their distribution after and before the rarefaction were reported (**Supplementary Figure 6**), confirming a sufficient sequencing depth for samples. The percentage and proportion of sequences in the analyzed cultivars (FR, LE, LA, OR) and conditions (T0, T1, T100, T200) is reported as a barplot (**Figure 2**), as well as information describing the identified taxa (**Supplementary Table 2**, **Figure 7**). Sequencing results at a phylum level indicate that the core microbiome associated to olive leaves included *Proteobacteria*, which accounted for 99.50% of total sequences (196.703), followed by *Firmicutes* (782 sequences, 0.39%), and *Actinobacteria* (208 sequences, 0.11%). Looking at the order level, the main bacterial orders are represented by *Burkholderiales* (86006 reads, 41.71%), *Enterobacterales* (66774 reads, 32.38%), and *Pseudomonadales* (42534 reads, 20.63%). Sequences distribution across the analyzed samples is reported in **Figure 2**. By comparing data of taxa abundance related to cultivar and treatment (**Figure 3**), consistent differences were observed only when moderate salt treatment was considered.

Looking at the cumulative abundance at the order level indicates that *Burkholderiale*s were mostly present in the T100 sample, with 34722 sequences compared to 14023 (T0), 18745 (T1), and 18516 (T200) reported in the other conditions. Conversely, the *Pseudomonadales* were well represented in all the experimental conditions (T0, 14276 sequences, T1 13594 sequences, T200 13585 sequences), except for T100 (1079 sequences).

### Impact of salt stress in microbial richness and composition

**Figure 4:**
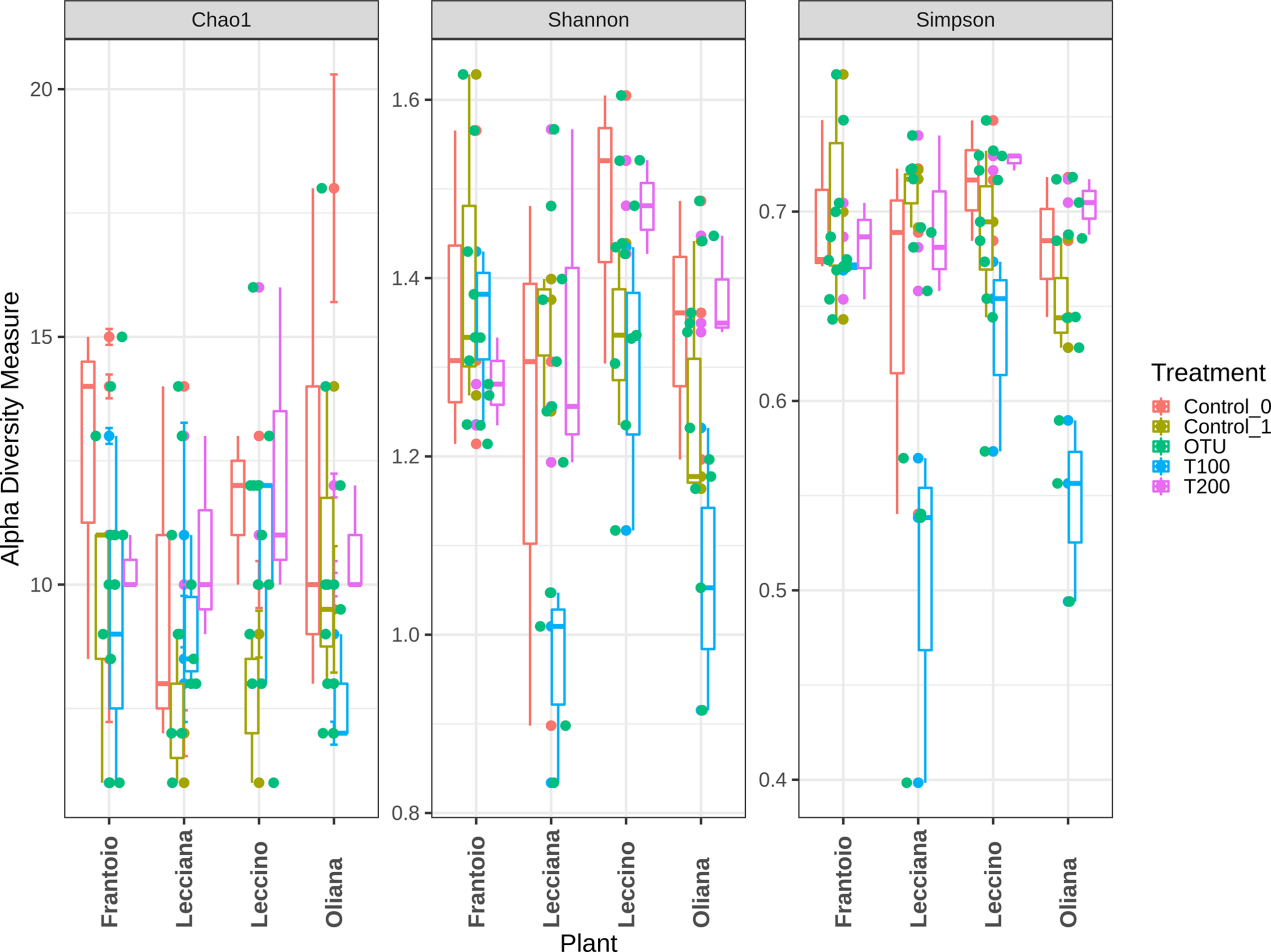
Alpha diversity assessment within analyzed samples. Data were computed based on three different indexes, Chao1, Shannon, and Simpson. Represented data show the diversity within each sample.

**Figure 5:**
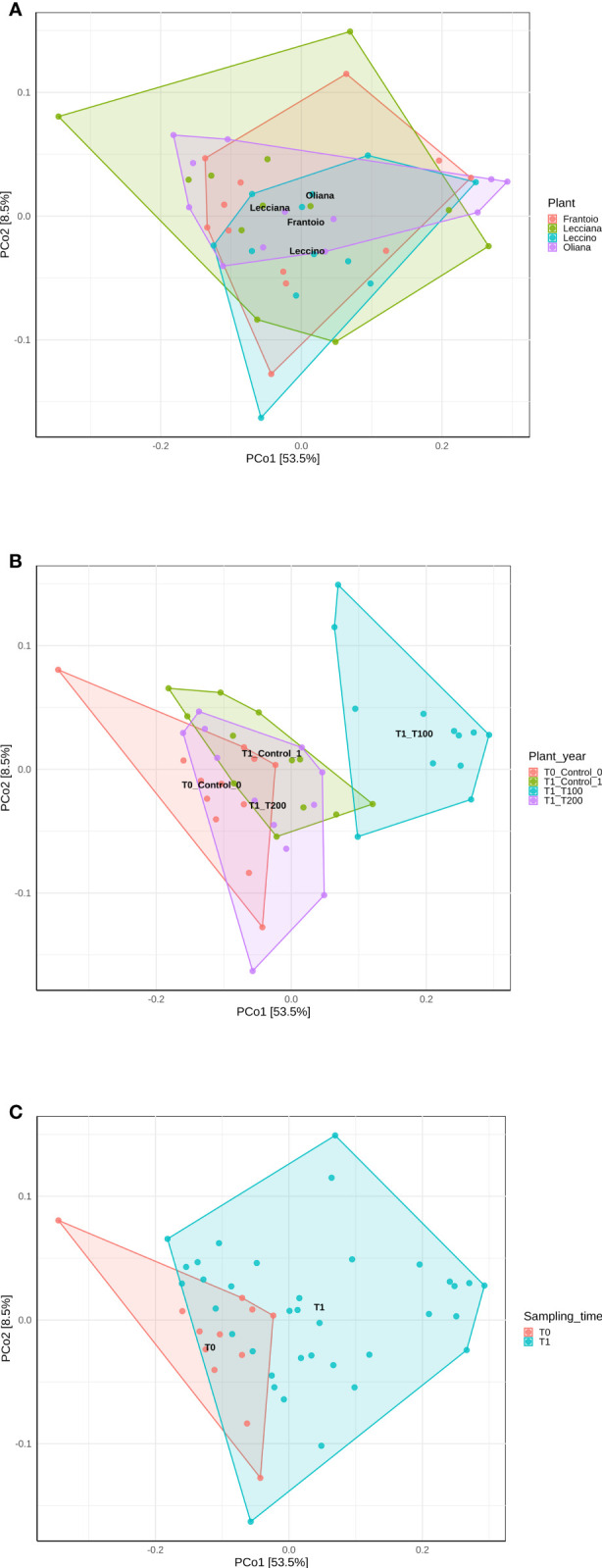
PCoA analyses of samples based on different variables using Bray-Curtis distance. Data were computed using the r package ampvis2, considering **(A)** cultivar, **(B)** treatment, and **(C)** sampling time. Total variance explained by each of the PCs is 62% (58.5% PC1, 8.5% PC2).

When cultivars were compared, we observed that differences in the LE endophytic microbiota did not occur in response to stress, according to the reported indexes. Conversely, alpha diversity indexes point out that bacterial community associated with OL and LA seems highly influenced by salt stress (**Figure 4**). Furthermore, differences mainly occurred in T100 than in T200. This is more evident by observing data from the Simpson index, where LA and OL reached a lower level of diversity in T100, whereas no other consistent differences were detected in the other samples (**Figure 5**). Under T200 treatment, the indexes increased reaching the control values, with the OL sample that increases the values of the Simpson and Shannon indexes. Beta-diversity was assessed according to PCoA analyses based on Bray-Curtis distances (**Figure 5**), which accounted for 62.0% of the total variance (53.5% PC1, 8.5% PC2). Results confirmed that samples clustered differently among the three considered variables (cultivar, treatment and sampling time). When the variable cultivar was considered, an overlap of the four cultivars was observed (**Figure 5A**).

**Figure 6:**
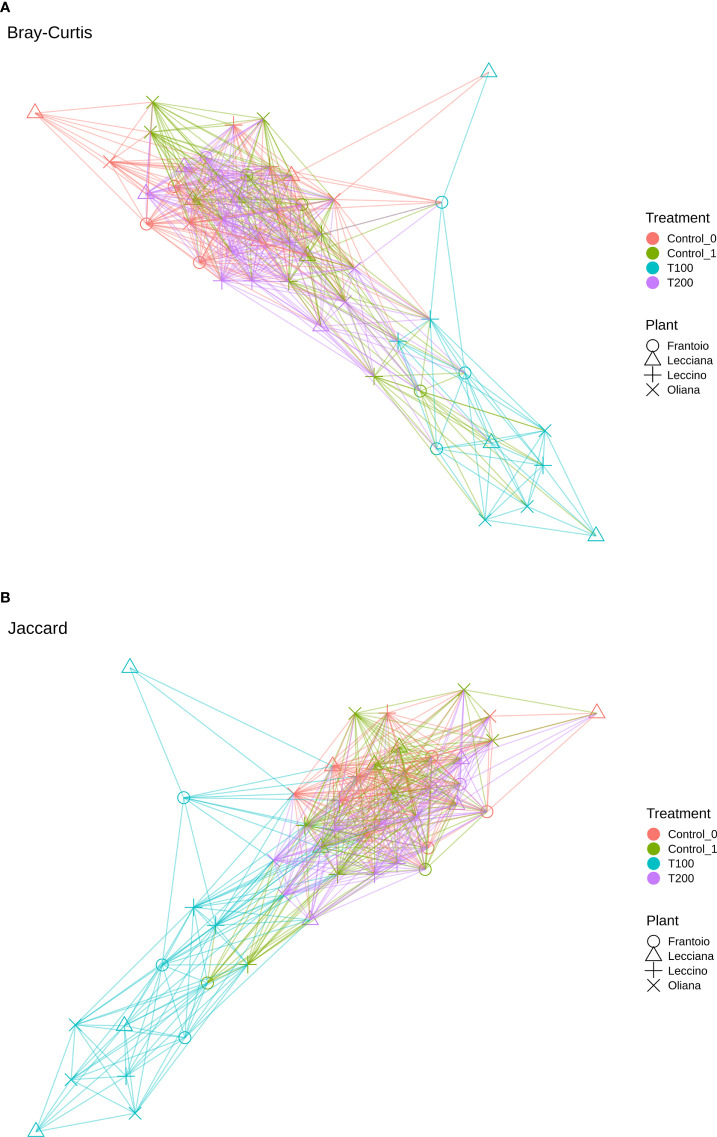
Network analysis based on Bray-Curtis **(A)** and Jaccard **(B)** coefficient.

The same scenario was also depicted in the case of the sampling time, as no significant differences were detected between the control T0 (sampling collected at the beginning of the experiment) and control T1 (**Figure 6C**), on the base of β-diversity, confirming that both can be considered as control samples. Conversely, when the treatment factor was considered, the T100 clustered separately from other treatment samples (**Figure 5B**), thus confirming the presence of distinctive features among the different conditions. Results from further multivariate analyses were reported in **Supplementary Figure 8**.

**Figure 7:**
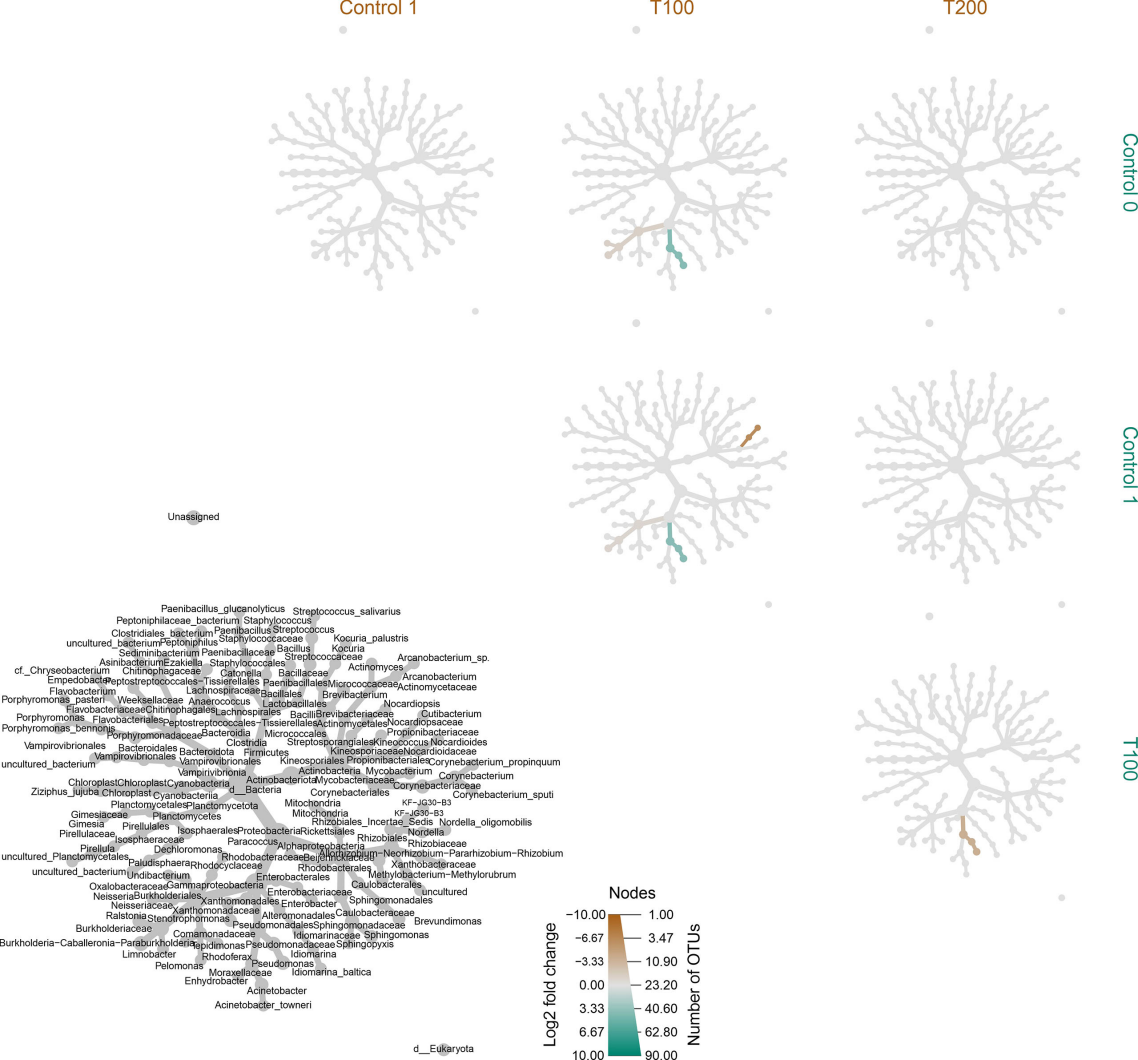
Differentially heat tree matrix for pairwise comparisons. Data were classified on FDR (cutoff = 0.05) and fold change using the r package metacoder (Foster et al., 2017) on samples classified based on treatment. The grey tree reported at the bottom left side works as a legend for the unlabeled trees. Each smaller tree represents a comparison between treatments in the columns and rows.

**Figure 8:**
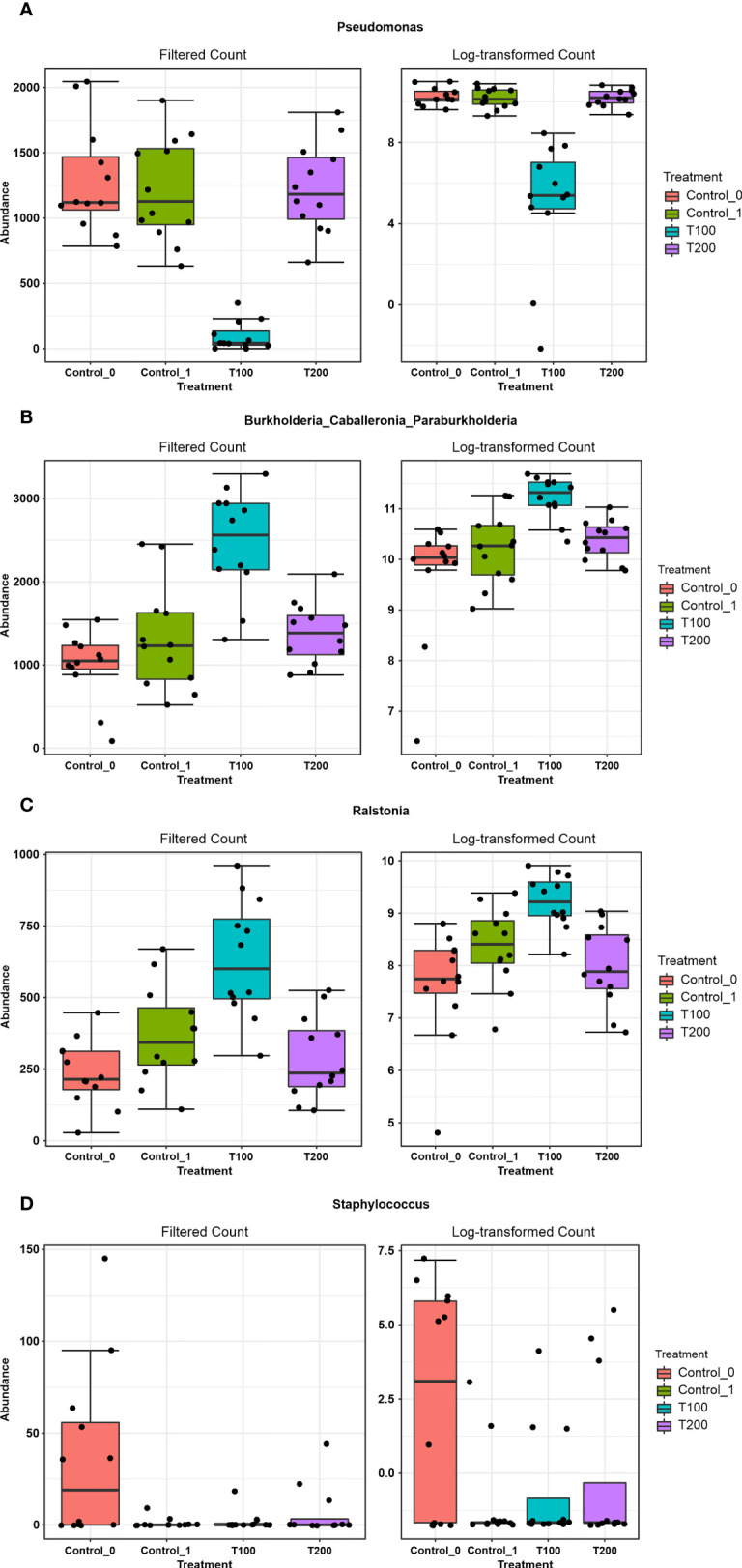
The outcome from Microbiome Analyst distinctive features among treatments. Boxplots represent the abundance (filtered and log-transformed count) of the taxa *Pseudomonas* **(A)**, *Burkholderia-Caballeronia-Paraburkholderia* **(B)**, *Ralstonia* **(C)** and *Staphylococcus* **(D)** in the four experimental conditions.

The differences in sample clusterization were confirmed by network analysis (**Figure 6**), where T100 resulted as clearly separated from other conditions, according to Bray Curtis and Jaccard indexes, leading to a distinct cluster group. Data reported in the differential heat tree (**Figure 7**) indicated that differences occurred in the pairwise comparison between T100 and the other considered conditions. Results from univariate analysis from MicrobiomeAnalyst (*p*-value ≤ 0.05) at genus level confirmed that four features were identified as significantly different among conditions, as reported in **Table 2**. The experimental conditions significantly altered the abundance of *Pseudomonas* (FDR = 1.36E^-11^), *Burkholderia* (FDR = 1.25E^-06^), *Ralstonia* (FDR = 2.02E^-05^) and *Staphylococcus* (FDR = 6.10E^-03^), as reported in **Figure 8**.

**Table 2:** Results of the univariate analysis on samples using the MicrobiomeAnalyst software.

Features	Pvalues	FDR	Statistics
***Pseudomonas***	1.23E-12	1.36E-11	4.00E+01
***Burkholderia_Caballeronia_Paraburkholderia***	2.28E-07	1.25E-06	1.66E+01
***Ralstonia***	5.52E-06	2.02E-05	1.23E+01
***Staphylococcus***	2.22E-03	6.10E-03	5.69E+00
*Enterobacter*	7.36E-02	1.38E-01	2.48E+00
*Undibacterium*	2.49E-01	3.91E-01	1.42E+00
*Paracoccus*	5.15E-01	6.43E-01	7.74E-01
*Cutibacterium*	5.40E-01	6.43E-01	7.30E-01
*Pelomonas*	5.84E-01	6.43E-01	6.55E-01
*Dechloromonas*	6.94E-01	6.94E-01	4.86E-01

Results indicate differentially abundant features sorted on False Discovery Rate (FDR, cutoff = 0.05). Data in bold are statistically significant.

The identification of statistically significant features was also performed through LEfSe analysis at the genus level, as reported in **Table 3**, which again confirmed that *Pseudomonas*, *Burkholderia* and *Ralstonia* represent the most notable features in the dataset. Data were then processed with the Random Forest algorithm, an ensemble of classification trees, each of which is grown by random feature selection from a bootstrap. Results reported in **Figure S9** show the cumulative error rates of random forest analysis including the different sample treatments. The Out-Of-Bug (OOB) error was 0.5.

**Table 3:** Result from LEfSe analysis at the genus level (FDR cutoff = 0.05).

Features	P values	FDR	Control_0	Control_1	T100	T200	LDAscore
***Pseudomonas***	6.36E-06	7.00E-05	750	667.83	50.5	686	2.54
***Burkholderia_Caballeronia_Paraburkholderia***	0.000132	0.000724	558.25	710.83	1322.4	768	2.58
***Ralstonia***	0.000246	0.000901	140.58	198.75	337.92	159.08	2
*Enterobacter*	0.036998	0.082156	978.75	862.25	715.5	822.33	2.12
*Staphylococcus*	0.037344	0.082156	20.083	0.41667	0.5	4.0833	1.03
*V6*	0.079102	0.1361	0.91667	4.8333	12.833	3.8333	0.843
*Cutibacterium*	0.086609	0.1361	1.8333	0	2.75	2.1667	0.376
*Paracoccus*	0.12929	0.17778	1.5833	0	3.0833	0.33333	0.405
*Undibacterium*	0.36684	0.44836	3	3.75	0.41667	0	0.459
*Pelomonas*	0.40802	0.44882	1.9167	8.8333	11.583	9.4167	0.766
*Dechloromonas*	0.91854	0.91854	2.0833	1.5	1.5	3.75	0.327

Differentially abundant features (treatments) were classified and sorted based on *p*-value and FDR. Data in bold are statistically significant.

## Discussion

The main goal of this study was to explore the changes in microbial endophytic communities in four different olive tree cultivars facing salt stress, under comparative experiments. Plants subjected to salt challenge showed peculiar differences in pigment and proline content compared to control ones, as well as in microbial endophytic communities, suggesting an effect of the stress on metabolite pathways. Interestingly, these data indicated that stress response appeared to have acted in a cultivar-dependent manner. For instance, Chla content showed no significant decrease in all cultivars. Conversely, data related to Chlb indicated a significant reduction in three out of four cultivars, reaching the lower level in T200. In FR, although not significant, a similar trend was also observed. A decrease in Chlb has been reported for other tree species under high salinity conditions (Ma et al., 1997; Yang et al., 2009). In olive, it has been suggested that reduction of chlorophyll content could be associated to oxidative stress processes and an increase in ROS scavenging enzymes (Regni et al., 2019). In our study, carotenoid content increased significantly mostly in FR cultivar and then, to a lesser extent, in OL. In olive tree, it has been suggested that carotenoid accumulation during stressed conditions could be related to metabolic changes occurring during stress adaptation, and the accumulation degree seems to be genotype-dependent (Bashir et al., 2021). Accordingly, it could be hypothesized that in FR cultivar the increase in salinity was accompanied by an enhancement of carotenoid synthesis withstand stress conditions, also confirming the status of FR as salt tolerant genotype (Rossi et al., 2015). On the other hand, the content of proline, which is known to favor homeostasis through osmotic regulation during salt stress (Di Martino et al., 2003; Iqbal et al., 2014), was not affected by salt stress on olive leaves, apart from a significant increase in a single cultivar (OL) under 200 mM salt stress. Our data partially contrasts with Abdallah et al. (2018) and Ben Ahmed et al. (2009), who found no evidence for an active role of proline in compensating osmotic unbalanced inside the leaf cells in different olive three cultivars, thereby suggesting that also the response of this osmolyte could be genotype-specific. However, it is worth noting that OL showed the lowest value of proline in T1 plants compared to the other cultivars. Moreover, as reported by Di Martino et al. (2003) in spinach, in addition to proline and glycine betaine, levels of free amino acids may change under a mild salt stress, with an enhancing in glycine and serine amounts. This result has been linked by these authors to an increase in photorespiration that might also have a role in the production of osmolytes. Although these aspects have not been investigated in this study, our results pave the way for further researches on how salt stress affect biochemical pathways in olive trees in response to salt stress.

Overall, analyzed olive tree leaves showed a limited, but specific taxa composition. It should be noted that endophytic bacteria may move into the plants by entering through primary roots and colonizing stems and leaves, or *via* stomata and lenticels present on leaves (Sørensen and Sessitsch, 2007; Compant et al., 2010). In the current study, the relative low richness in taxa could be potentially related to growing conditions, where the presence of an inert substrate like perlite could have hampered the own plant’s possibility to establish an efficient endophytic community. It is in fact possible to hypothize that using a natural soil, where microbial communities generally show an high biodiversity, the endophytic bacterial communities in leaves could reflect the complexity present in soil (Malacrinò et al., 2022). The bacterial communities inhabiting the leaves of all cultivars included members belonging to three main phyla, *i.e.*, *Proteobacteria*, *Actinobacteria* and *Firmicutes*. The most abundant class was composed by *Gammaproteobacteria*, as also observed in previous studies on the phyllosphere of other olive tree cultivars (Mina et al., 2020). Not surprising, *Actinobacteria* and *Gammaproteobacteria* were found to be common inhabitants of angiosperm leaves (Redford et al., 2010). At family level, results also confirmed that *Pseudomonas* represents one of the main taxa living inside the olive tree leaves, as already demonstrated (Vergine et al., 2019). *Pseudomonas* spp. is an important group of bacteria known to be able to improve salt stress tolerance of plants. It was demonstrated that inoculation of *Pseudomonas* improves growth and salt-tolerance in different plants, such as rice (Jha et al., 2011), mustard (Phour and Sindhu, 2020), cotton (Yao et al., 2010), cucumber (Gamalero et al., 2010), and canola (Cheng et al., 2012). It is worth noting that the core microbiome detected in the four olive cultivars was similar to that already observed for other olive tree cultivars (Mina et al., 2020), suggesting that this species may possess a species-specific resident microbiome in the phyllosphere, which is shared by the considered different genotypes/cultivars. At genus level, in addition to *Pseudomonas*, *Ralstonia* and *Burkholderia*, also *Enterobacter* ssp. and *Pelomonas* spp. were detected. These two genera were often observed in olive phyllosphere (Müller et al., 2015; Mina et al., 2020) and it should be noted that *Pseudomonas*, *Ralstonia* and *Pelomonas* are known to represent three main genera of the olive phyllosphere core microbiome in European cultivars (Müller et al., 2015). Intriguingly, it has been previously observed that olive root bacterial communities appeared stable in taxa composition across genotypes and seasons (Chialva et al., 2021). However, Mina and colleagues (2020) observed significant differences between microbial composition of phyllosphere of two Spanish olive cultivars grow in orchards (Mina et al., 2020). In the current study, the absence of significant differences in microbial communities among the considered cultivars may be explained by the sterile substrate used for growing plants that might have limited the bacterial recruitment by roots from actual soil.

A shift in endophytic resident bacterial community inside the leaves during salt exposure was detected by the metabarcoding approach applied on the olive tree phyllosphere. A significant change in the bacterial community under T100 treatment was observed. In this regard, it could be assumed that this stress level allowed to maintain an active transport of nutrients, water, and sodium inside the leaves, which may have favored the shift of endophytes towards specific bacteria taxa able to survive in this cellular context. Particularly, *Burkholderia* and *Ralstonia*, both belonging to *Burkholderiales* order, increased under this salt stress condition. Species belonging to *Burkholderia* genus are known to be able to survive under high level of salinity and to enhance plant tolerance to salt stress (Eberl and Vandamme, 2016; Yang et al., 2020). The genus *Burkholderia* contains more than 62 species, among which some are pathogenic, and others play a mutualistic role in plants. Several studies have described the importance of *Burkholderia* against abiotic stress in plants (Estrada-De Los Santos et al., 2001; Suárez-Moreno et al., 2012; Theocharis et al., 2012). For instance the inoculation of *B. phytofirmans* on quinoa plants irrigated with saline water increased growth rate and mitigated osmotic stress through an osmosis compensation and increased production of catalase enzymes (Yang et al., 2020). In addition, the mutualistic role of *B. phytofirmans*, enhancing tolerance against abiotic stress through a priming acclimation process has been also demonstrated in grapevine (Theocharis et al., 2012). Furthermore, *Burkholderia* was identified as differentially regulated taxa in a study describing the role of the olive tree bacterial communities present in soil and tissues (leaf surface and xylem sap) under a sustainable or conventional orchard, with a strong prevalence for the latter management condition (Fausto et al., 2018). Additionally, *Burkholderia* species are recognized to be able to reduce ethylene levels by ACC deaminase, one of the main processes that help plant growth in environmental stressed conditions (Sarkar et al., 2018). The enhancement of *Burkholderia* endophytic taxa in 100 mM NaCl-treated plants over the main genus found in olive leaves, *i.e.*, *Pseudomonas*, suggested an ecological shift of the native olive endophtyic microbiome, triggered at cellular level by osmotic conditions. The decrease of *Pseudomonas* taxa showed also a secondary effect that include the increase in abundance of antagonistic *Ralstonia* species, as previously observed (Chandrasekaran et al., 2016). This genus has been reported to include plant pathogenic species, as well as species resistant to stressful conditions and disturbed environments (Marques et al., 2010).

Interestingly, the bacterial community did not change in the highest salt treatment (T200) compared with the control, suggesting no effect on the shaping of salt tolerant olive tree microbiome under high level of salinity. In this context, it has already been reported that moderate levels of salinity can favor bacterial diversity of soil microbial communities; conversely, bacteria communities are rapidly replaced by fungal ones at high salinity levels (Tufail et al., 2021). A role in boosting salt tolerance by the phyllosphere microbiome has been proposed so far, as in the case of leaves of some halophytic plants that harbor halotolerant and extreme halophiles bacterial species (Mora-Ruiz et al., 2015). Additionally, olive trees exposed to salt stress are known to activate adaptation processes, also including cytosine methylation affecting regulation of gene expression at transcriptional and post-transcriptional level (Mousavi et al., 2019). In addition, it has been suggested that plants modulate the regulation of microRNA (miRNA) to favor the association with beneficial endophytic bacteria by suppressing defense processes (Carvalho et al., 2016). In our experiment, we cannot exclude that these epigenetic mechanisms may have played a role in both adaptations of plants to moderate salt stress conditions and on the selection of bacterial population under T100 salt treatment. It is possible to speculate that adaptation through epigenetic processes may contribute to explain why under T200 salt treatment, plants did not change bacterial endophytic community, possibly due to epigenetic and microbiota interactions (Vannier et al., 2015). Although this experiment did not provide any data on this aspect, this intriguing hypothesis deserved to be explored in further experiments in the same olive tree cultivars.

In the whole and according to results from biochemical analysis, two cultivars, *i.e.*, FR and OL, can be considered as salt tolerant, while LE and LA were found to be salt sensitive. Metabarcoding data demonstrated that resident endophytic microbiome was affected by salt stress, but no significant differences in microbial abundance and richness were observed among cultivars. Taken together, these results suggested that, at least upon these experimental growth conditions (sterilized substrate), the genetic and/or epigenetic background of olive trees may play a primary role in salt tolerance, whereas the detected shift in endophytic microbiome seems to be a common secondary process. However, a diverse priority of these processes in more complex natural conditions cannot be excluded.

## Conclusion

The integration of data from biochemical analyses and assessment of microbial endophytic communities in olive trees was a suitable approach to evaluate the tolerance of cultivars to environmental challenges. In the present study, a characterization of changes of endophytic resident microbiota profiles in olive trees subjected to salt stress was performed using an NGS-based approach. Different salt concentrations affected the leaf endophytic bacterial composition, highlighting that plant system is strongly connected with the microbial community inside them; this condition was evident for plants treated with 100 mM NaCl. In this case, data demonstrated the enrichment of a peculiar endophytic community, which could play a significant role in the ability of olive genotypes to withstand salt stress. Under this perspective, the abundance of taxa like *Burkholderia* in this experimental condition could reinforce the hypothesis that the endophytic community may play a role in helping olive trees to deal with the harmful effects of sodium inside the leaves. Conversely, species of the *Pseudomonas* genus, the main taxon of native olive leaves microbiome, seem to have an opposite trend, reinforcing the hypothesis of an ecological shift towards tolerant bacterial strains. These data offer a comprehensive adaption scheme of olive trees cultivar to salt stress, deepening plant-microbial interactions and thus representing an innovative starting point for the constitution and management of new cultivars adapted to salt stress.

## Data availability statement

The data presented in the study are deposited in the Figshare repository. Accession number(s) can be found below: https://doi.org/10.6084/m9.figshare.20291937.

## Author contributions

FV and LS designed the research. MV performed the DNA extraction. FV, FS and SG performed the bioinformatic and statistical analyses. WGN and LS performed the pigment and proline analyses. FV, FS, RB, ASc, and ASa drafted the paper. MV, AL, WGN, LDB, and SM helped to revise the manuscript with all authors contributing to the discussion of the data. FV, AL and SM provided funds for the research. All authors contributed to the article and approved the submitted version.

## Funding

This study was partially supported with funds for the project PON Ricerca ed Innovazione 2014-2020, azione II, PON ARS01_01136 “E-Crops – Tecnologie per un’Agricoltura Digitale e Sostenibile” to ASc. This project was supported by the Fondazione Caripit, Grant/Award Number: 2018.0527 to FV.

## Conflict of interest

The authors declare that the research was conducted in the absence of any commercial or financial relationships that could be construed as a potential conflict of interest.

## Publisher’s note

All claims expressed in this article are solely those of the authors and do not necessarily represent those of their affiliated organizations, or those of the publisher, the editors and the reviewers. Any product that may be evaluated in this article, or claim that may be made by its manufacturer, is not guaranteed or endorsed by the publisher.

## Supplementary material

Supplementary Figure 1 Representative image of the experimental design.

Supplementary Figure 2 Necrotic damage caused by saline stress (T200, Frantoio).

Supplementary Figure 3 The olive tree plants’ biometric results (plant height and total number of leaves) at two different sampling points (T0 and T1).

Supplementary Figure 4 Representative plants grouped by cultivar collected at the end of the experiment.

Supplementary Figure 5 Results of Chla/Chlb ratio.

Supplementary Figure 6 Results of rarefaction on samples grouped according to cultivar. Curves are getting before **(A)** and after **(B)** rarefaction processes. The horizontal axis indicates the sequences resulting from the Illumina MiSeq sequencing platform on V3-V4 regions. The vertical axis shows the number of amplified sequence variants (ASVs).

Supplementary Figure 7 ASV circle computed on samples classified based on **(A)** treatment and **(B)** cultivar. Min_prop_tax parameter = 0.005, data computed at Family level.

Supplementary Figure 8 Data ordination using different multivariate methods. CCA, canonical correspondence analysis; DCA, Detrended correspondence analysis; DPCoA, Double Principle Coordinate Analysis; NMDS, Non-metric multidimensional scaling; PCoA, Principal Coordinate Analysis; RDA, Redundancy analysis.

Supplementary Figure 9 The classification of cumulative error rates by Random Forest (Liaw et al., 2002). The overall error rate is shown as the black line; the red and green lines represent the error rates for each class. Random forest is an example of supervised machine learning algorithm that can be used to identify a specific subset of microbial taxa able to predict a target variable according to relative abundances.
