# Supplementary material for: Salt stress in olive tree shapes resident endophytic microbiota
Source: Front Plant Sci. 2022 Sep 29;13:992395. doi: 10.3389/fpls.2022.992395 (PMC9556989; doi:10.3389/fpls.2022.992395)
Supplement: Supplementary file 1 [file Table_1.docx]

**Table S2.** Post-hoc test results of two-way ANOVA analyses on Chl a/b ratio.

| *Tukey's multiple comparisons test* | *Predicted (LS) mean diff,* | *95,00% CI** | *Significant?* | *Summary* | *Adjusted P-Value* |
| --- | --- | --- | --- | --- | --- |
| ***Chlorophyll a/b ratio*** |  |  |  |  |  |
| **FR** |  |  |  |  |  |
| 0 mM vs. 100mM | -0,01155 | -1,037 to 1,014 | No | ns | >0,9999 |
| 0 mM vs. 200mM | -1,593 | -2,664 to -0,5226 | Yes | *** | 0,0003 |
| 100mM vs. 200mM | -1,582 | -2,607 to -0,5567 | Yes | *** | 0,0002 |
| **LA** |  |  |  |  |  |
| 0 mM vs. 100mM | -0,9273 | -2,020 to 0,1654 | No | ns | 0,1702 |
| 0 mM vs. 200mM | -1,794 | -2,887 to -0,7012 | Yes | **** | <0,0001 |
| 100mM vs. 200mM | -0,8667 | -1,844 to 0,1107 | No | ns | 0,1281 |
| **LE** |  |  |  |  |  |
| 0 mM vs. 100mM | -1,631 | -2,724 to -0,5386 | Yes | *** | 0,0003 |
| 0 mM vs. 200mM | -1,762 | -2,854 to -0,6690 | Yes | **** | <0,0001 |
| 100mM vs. 200mM | -0,1304 | -1,108 to 0,8470 | No | ns | >0,9999 |
| **OL** |  |  |  |  |  |
| 0 mM vs. 100mM | -2,036 | -3,128 to -0,9430 | Yes | **** | <0,0001 |
| 0 mM vs. 200mM | -1,629 | -2,722 to -0,5366 | Yes | *** | 0,0003 |
| 100mM vs. 200mM | 0,4064 | -0,5710 to 1,384 | No | ns | 0,9536 |
