# Supplementary figures and images for: Salt stress in olive tree shapes resident endophytic microbiota
Source: Front Plant Sci. 2022 Sep 29;13:992395. doi: 10.3389/fpls.2022.992395 (PMC9556989; doi:10.3389/fpls.2022.992395)

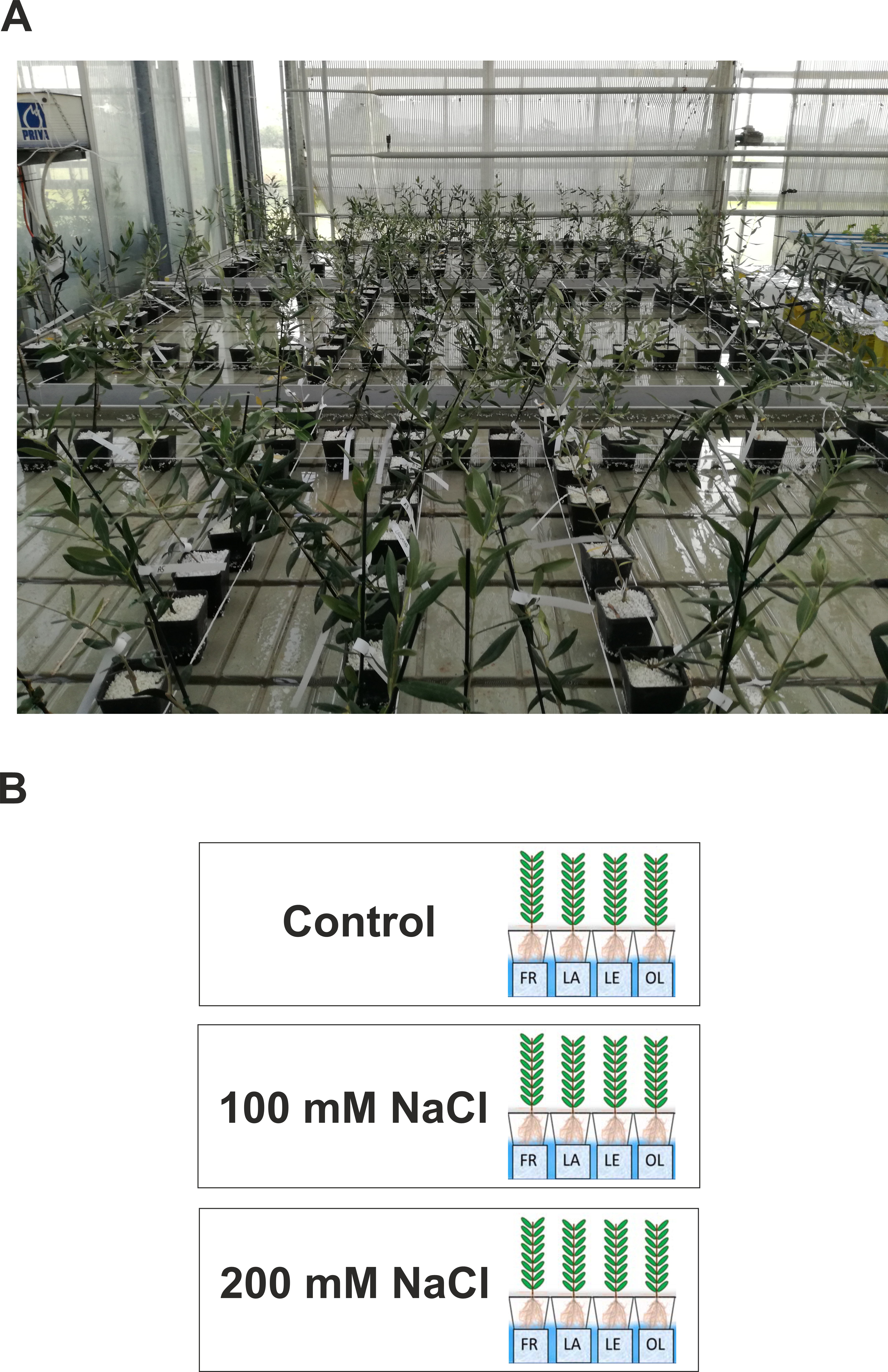

Supplement: Supplementary Figure 1 — Representative image of the experimental design. [file Image_1.jpeg]

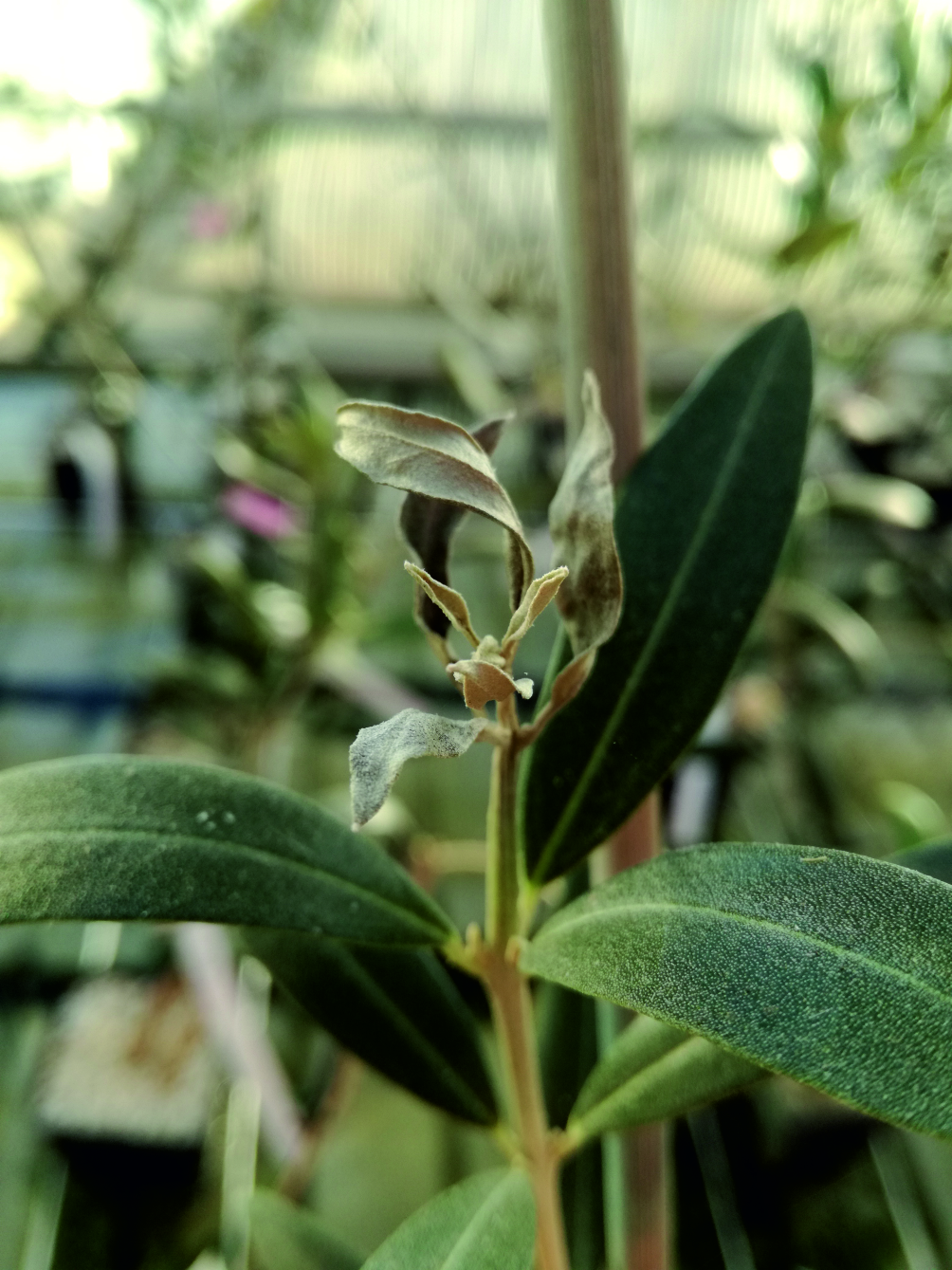

Supplement: Supplementary Figure 2 — Necrotic damage caused by saline stress (T200, Frantoio). [file Image_2.tif]

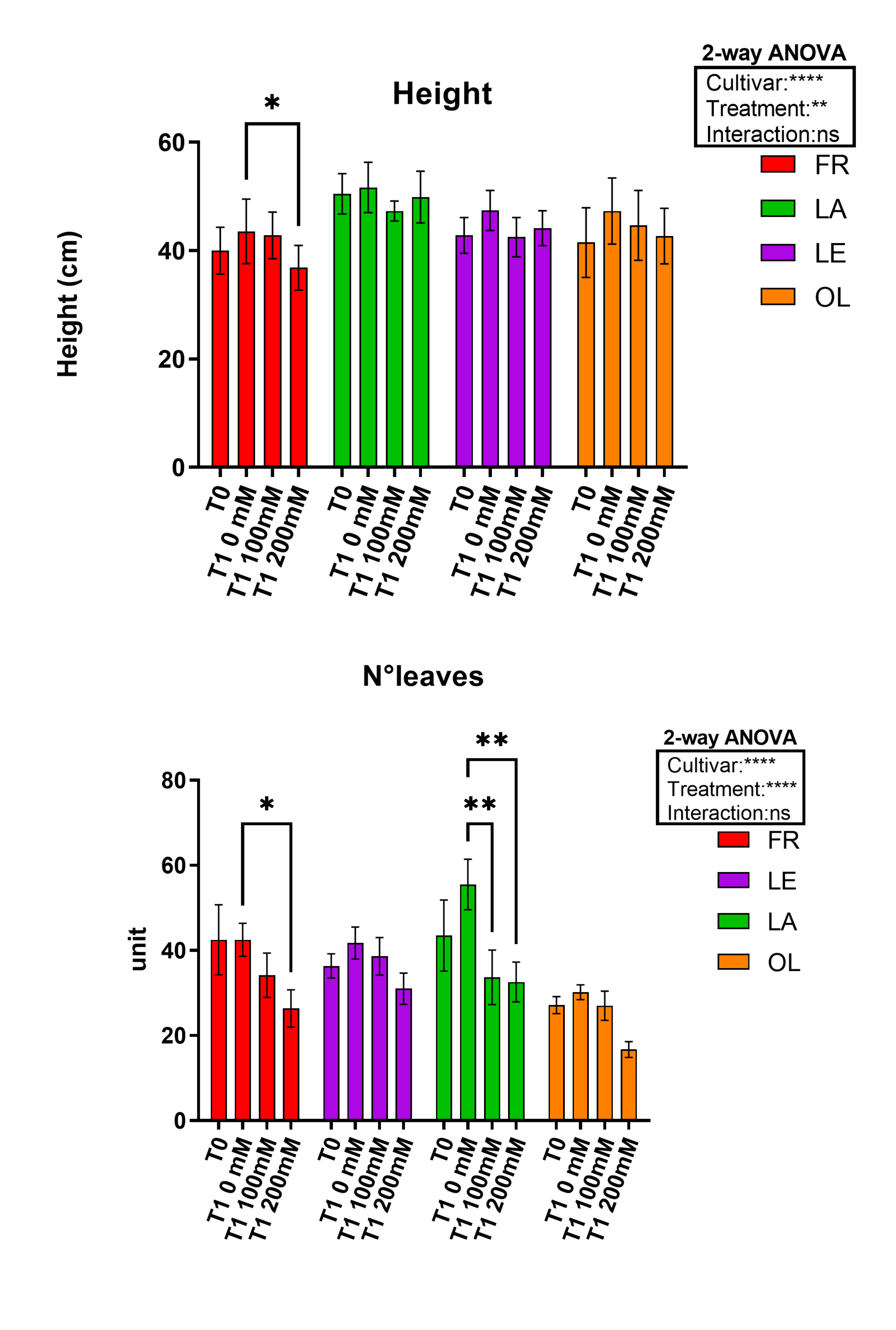

Supplement: Supplementary Figure 3 — The olive tree plants’ biometric results (plant height and total number of leaves) at two different sampling points (T0 and T1). [file Image_3.tif]

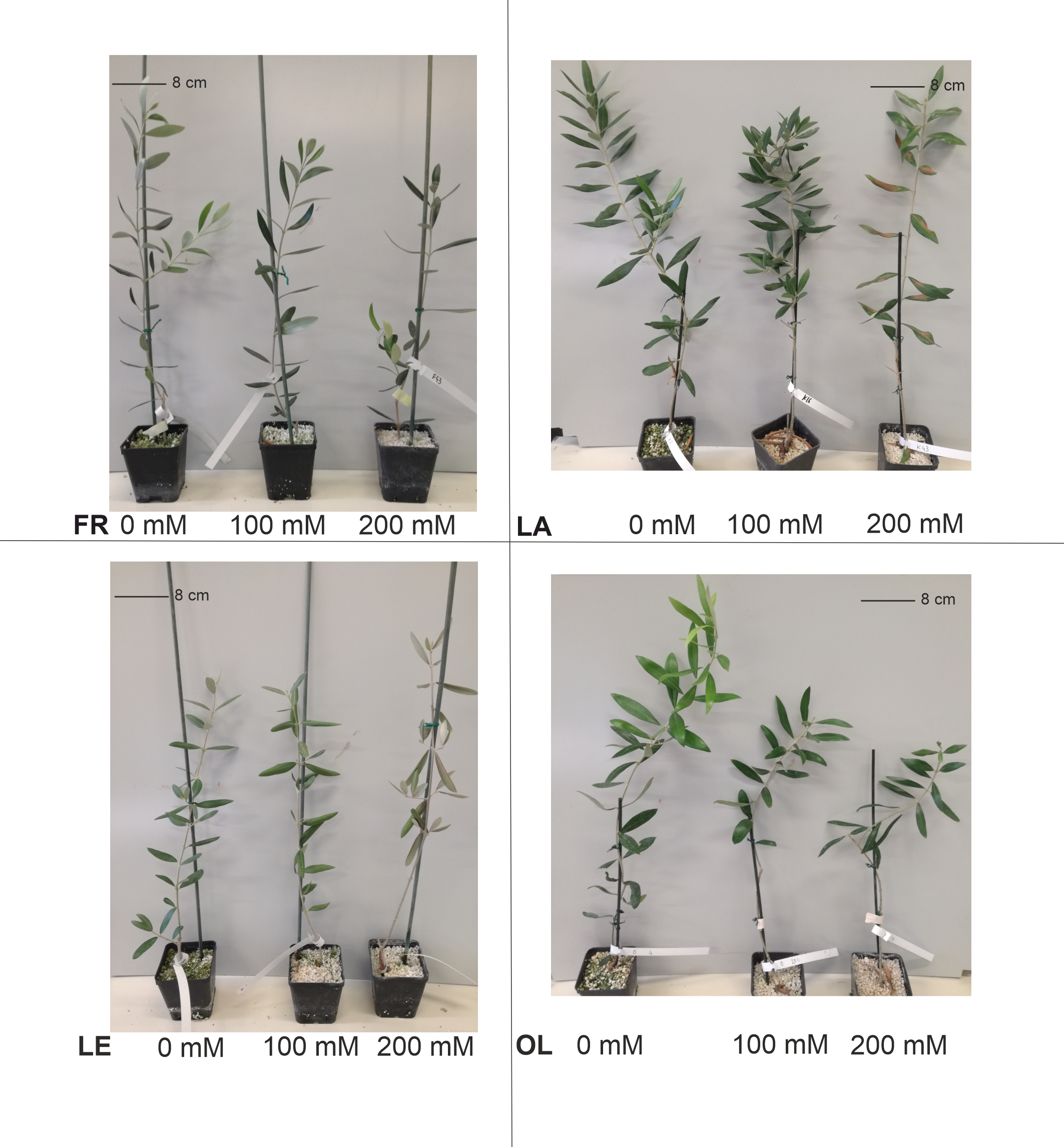

Supplement: Supplementary Figure 4 — Representative plants grouped by cultivar collected at the end of the experiment. [file Image_4.tif]

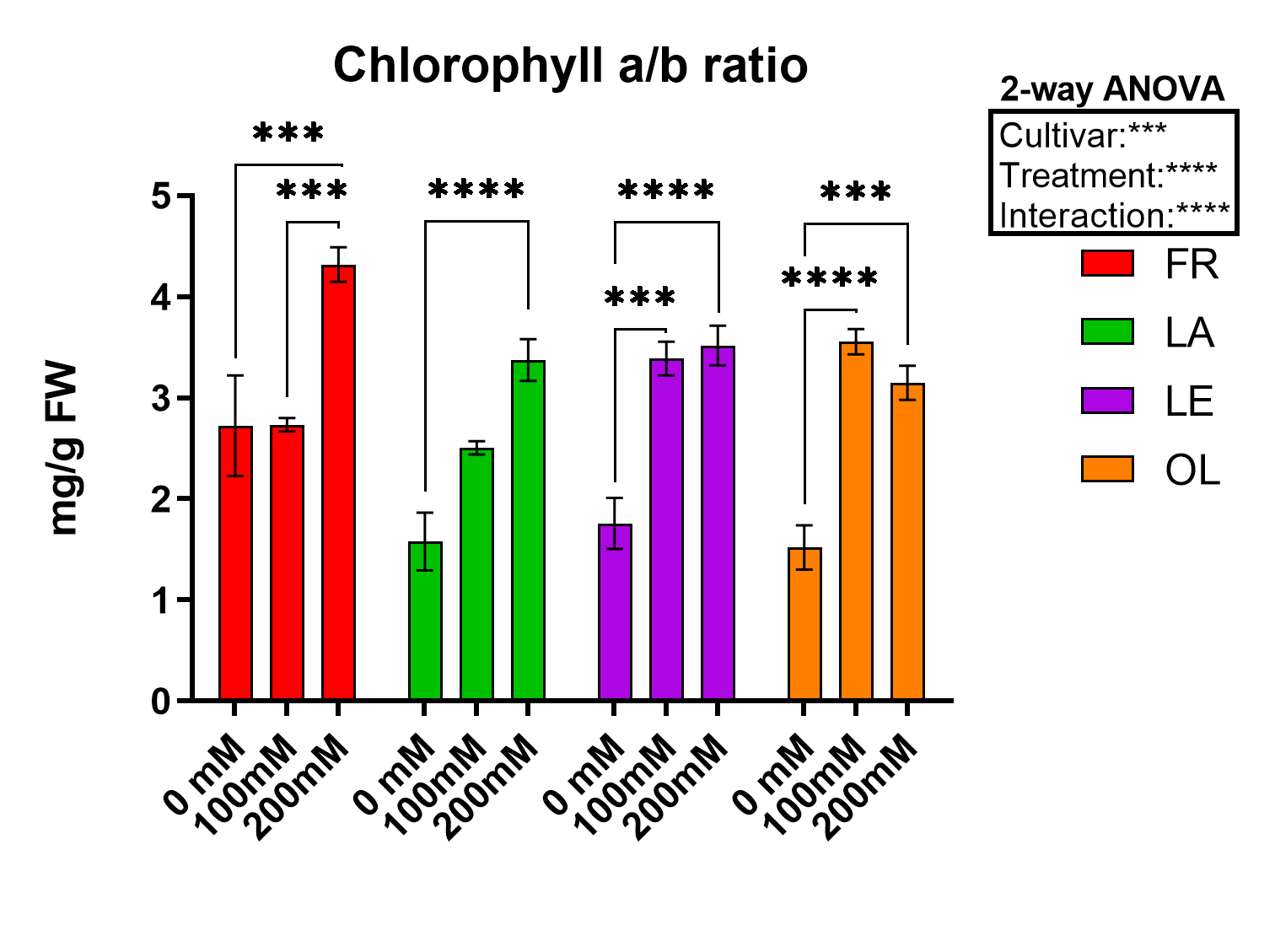

Supplement: Supplementary Figure 5 — Results of Chla/Chlb ratio. [file Image_5.tif]

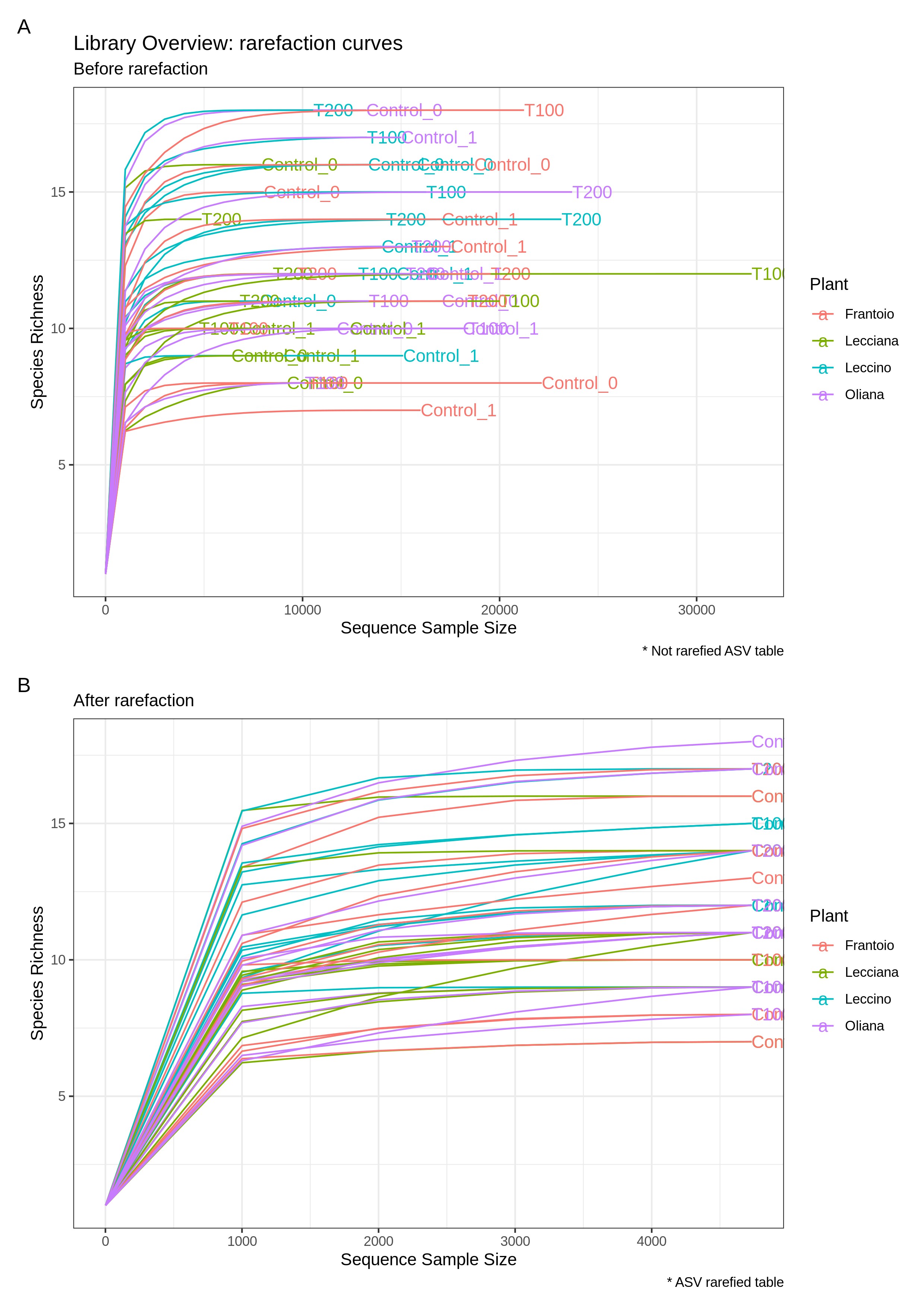

Supplement: Supplementary Figure 6 — Results of rarefaction on samples grouped according to cultivar. Curves are getting before (A) and after (B) rarefaction processes. The horizontal axis indicates the sequences resulting from the Illumina MiSeq sequencing platform on V3-V4 regions. The vertical axis shows the number of amplified sequence variants (ASVs). [file Image_6.jpeg]

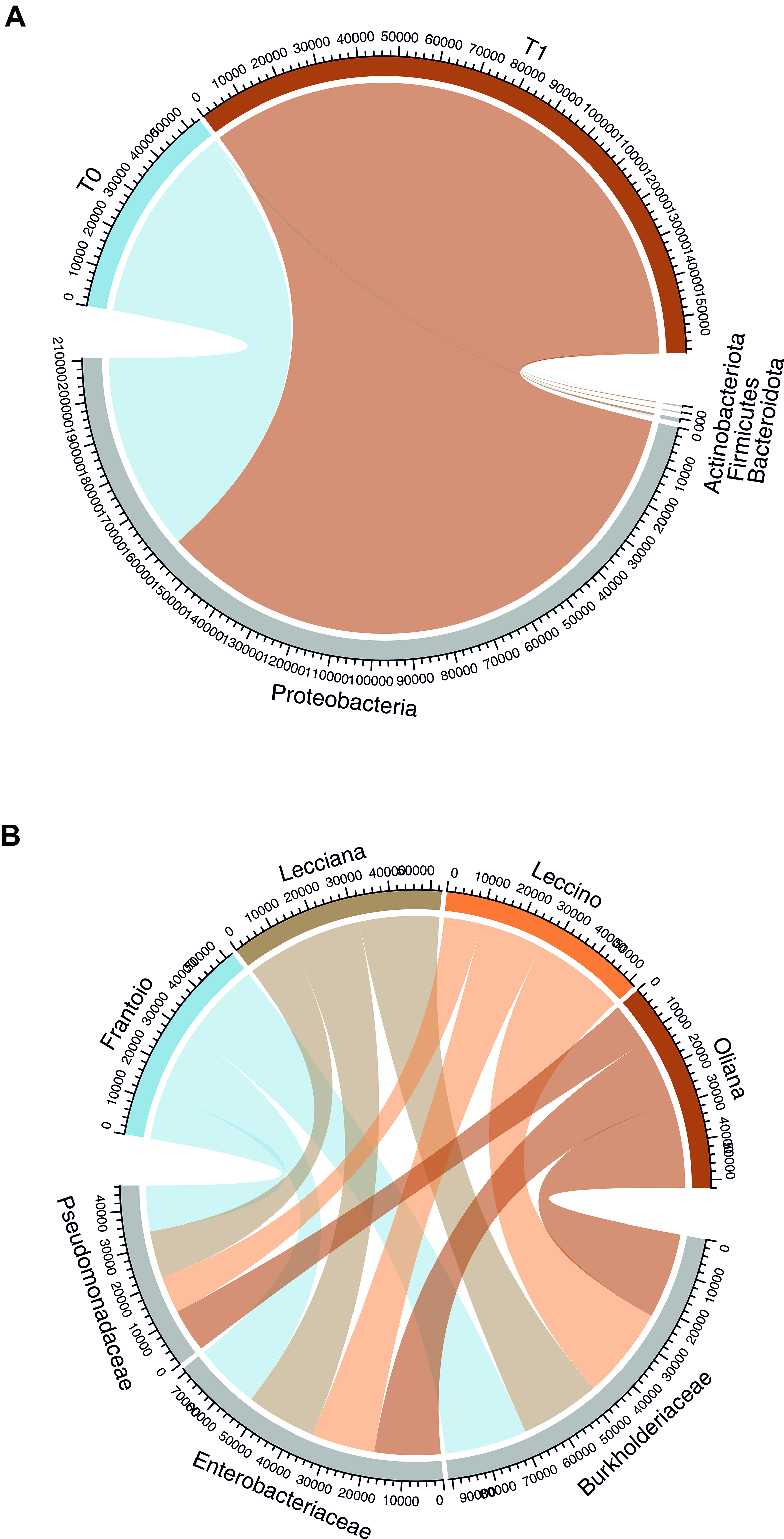

Supplement: Supplementary Figure 7 — ASV circle computed on samples classified based on (A) treatment and (B) cultivar. Min_prop_tax parameter = 0.005, data computed at Family level. [file Image_7.tif]

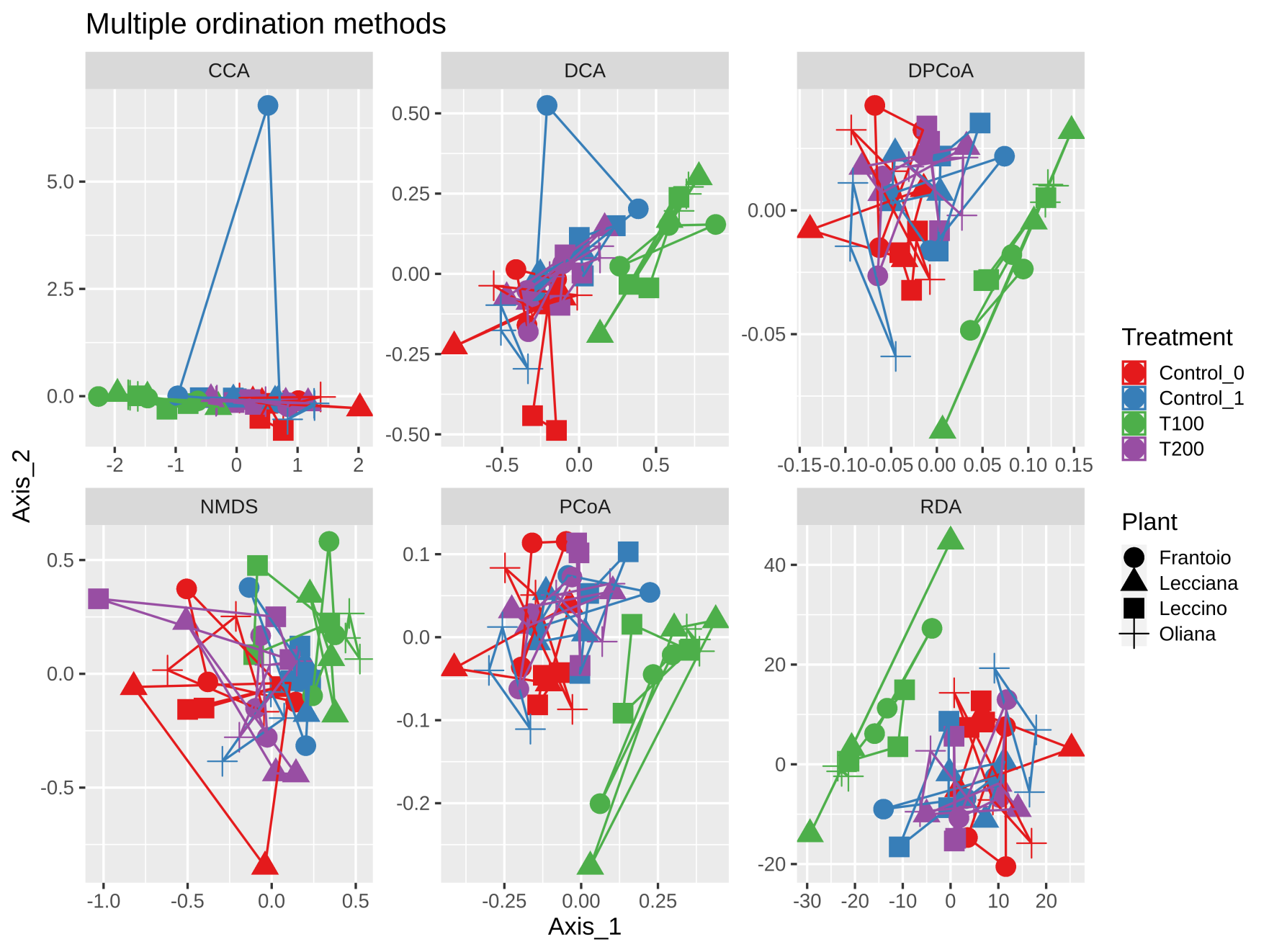

Supplement: Supplementary Figure 8 — Data ordination using different multivariate methods. CCA, canonical correspondence analysis; DCA, Detrended correspondence analysis; DPCoA, Double Principle Coordinate Analysis; NMDS, Non-metric multidimensional scaling; PCoA, Principal Coordinate Analysis; RDA, Redundancy analysis. [file Image_8.tif]

# Random Forest Classification

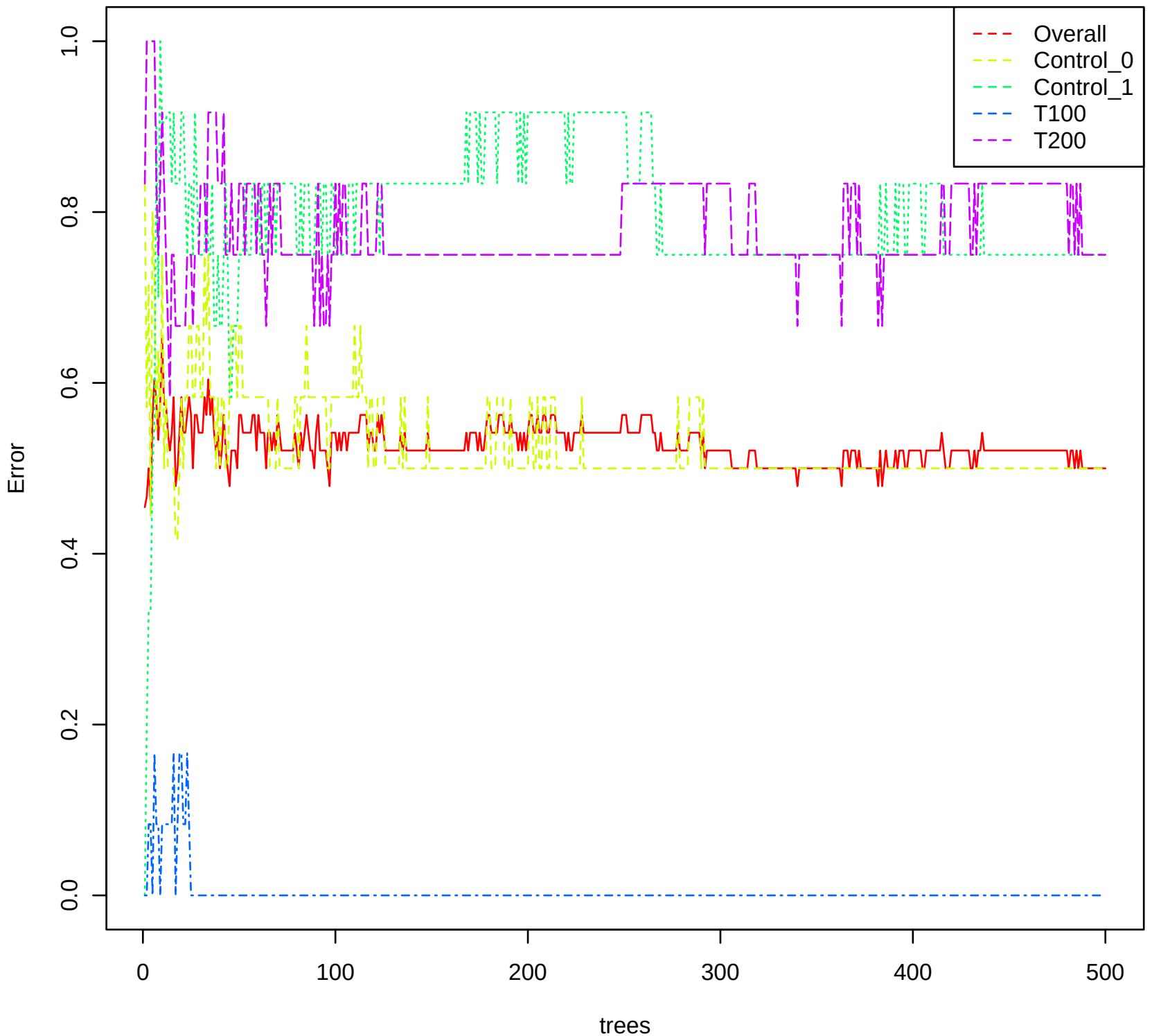

Supplement: Supplementary Figure 9 — The classification of cumulative error rates by Random Forest (Liaw et al., 2002). The overall error rate is shown as the black line; the red and green lines represent the error rates for each class. Random forest is an example of supervised machine learning algorithm that can be used to identify a specific subset of microbial taxa able to predict a target variable according to relative abundances. [file DataSheet_1.pdf]
